# Systematic Bandgap Engineering of a 2D Organic–Inorganic Chalcogenide Semiconductor via Ligand Modification

**DOI:** 10.1021/jacs.5c07989

**Published:** 2025-08-19

**Authors:** Tomoaki Sakurada, Watcharaphol Paritmongkol, Yeongsu Cho, Woo Seok Lee, Petcharaphorn Chatsiri, Julius J. Oppenheim, Ruomeng Wan, Annlin Su, Nicholas Samulewicz, Khemika Wannakan, Peter Müller, Mircea Dincă, Heather J. Kulik, William A. Tisdale

**Affiliations:** † Department of Chemical Engineering, Massachusetts Institute of Technology, Cambridge, Massachusetts 02139, United States; ‡ Department of Materials Science and Engineering, School of Molecular Science and Engineering, 423058 Vidyasirimedhi Institute of Science and Technology (VISTEC), Rayong 21210, Thailand; § Department of Materials Science and Engineering, Massachusetts Institute of Technology, Cambridge, Massachusetts 02139, United States; ⊥ Department of Chemical and Biomolecular Engineering, School of Energy Science and Engineering, Vidyasirimedhi Institute of Science and Technology (VISTEC), Rayong 21210, Thailand; ¶ Department of Chemistry, 2167 Massachusetts Institute of Technology, Cambridge, Massachusetts 02139, United States

## Abstract

Hybrid organic–inorganic semiconductors present new opportunities for optoelectronic materials design not available in all-organic or all-inorganic materials. One example is silver phenylselenide (AgSePh) – or “mithrene” – a blue-emitting 2D organic–inorganic semiconductor exhibiting strong optical and electronic anisotropy. Here, we show that the bandgap of mithrene can be systematically tuned by introducing electron-donating and electron-withdrawing groups to the phenyl ligands. We synthesized nine mithrene variants, eight of which formed 2D van der Waals crystals analogous to those of AgSePh. Density functional theory calculations reveal that these 2D mithrene variants are direct-gap or nearly direct gap semiconductors. Furthermore, we identify correlations between the optical gap and three experimental observables – the Hammett constant, ^77^Se chemical shift, and selenium partial charge – offering predictive power for bandgap tuning. These findings highlight new opportunities for applying the tools of chemical synthesis to semiconductor materials design.

## Graphical abstract

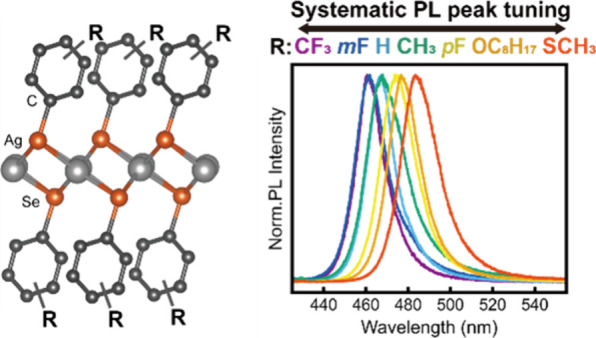

## Introduction

The choice of semiconductor material for many applications is determined by its electronic bandgap. In conventional inorganic semiconductors, bandgap tuning is achieved through mixing, alloying, or doping of its elemental composition. For organic semiconductors, by contrast, bandgap tuning is accomplished through molecular engineering techniques such as functionalization and conjugated system expansion. In hybrid organic–inorganic semiconductors, one of these approaches is usually more impactful than the other – that is, materials behavior is predominantly influenced by either inorganic or organic modification alone. For example, in hybrid organic–inorganic halide perovskites, the bandgap is primarily affected by the inorganic framework,^1^ with minimal impact from modifications to the organic components unless accompanied by structural changes.^2–4^ This occurs because, in halide perovskites, the organic moieties mainly act as structure-directing agents without participating in frontier orbitals.^5^

Metal organochalcogenides (MOCs) are a family of hybrid organic–inorganic materials that have regained interests in recent years due to the discovery of their semiconducting properties.^6–9^ MOCs display many promising characteristics, including bright luminescence,^10–12^ carrier transport,^13,14^ giant optical anisotropy,^15,16^ ultrastrong light-matter coupling,^17^ and chemical robustness.^8^ As a result, they have been investigated for use as light emitters,^10,11,18–21^ photoconductors,^22–24^ sensors,^12,25^ and electro- and photocatalysts.^26,27^ Among the most promising MOCs is silver phenylselenide (AgSePh), a blue emitting MOC known also as mithrene,^18^ and its derivatives.^28–30^

Previous research on a variety of MOC materials has shown promise for bandgap tuning by changing the inorganic or organic composition. For example, Demessence and co-workers showed optical property tuning in one-dimensional (1D) thiol MOCs via metal substitution.^31^ Li et al. demonstrated electronic band gap tuning of silver phenylsulfide through metal substitution and phenylthiolate derivatization.^32^ Yang et al. studied dimensional engineering of Pb-based MOCs by substituting the *para*-functionalization of phenylthiolate ligands, achieving tunable electronic and optical properties.^21^ A few research teams also reported chalcogen substitution in silver phenylchalcogenides, resulting in changing optical absorption of the materials from ultraviolet to visible.^20,30,33,34^ However, a systematic demonstration of optical emission tuning by ligand modification and accompanying mechanistic understanding of this behavior, is lacking.

In this work, we report an experimental investigation of the underlying mechanism of optical gap tuning in mithrene by organic modification. AgSePh – or mithrene – is the most widely studied member of the MOC family,^16,18,20,23,29,33–35^ and a strong candidate for use in optoelectronic devices. We synthesized eight analogues of AgSePh, six of which are novel compounds, by functionalizing the phenyl ring with small electron-withdrawing or electron-donating groups to tailor electronic contribution with minimal change in the steric factor. We found that the Ag–Se cores of these MOCs are mostly maintained and their photoluminescence (PL) emission peaks could be tuned from 461 to 486 nm. Density functional theory (DFT) calculations were performed, revealing that the organic functional groups modulate the electron density near the selenium atoms. Finally, we present correlations between the optical gap and three experimental observables – the Hammett constant of the organic functional group, ^77^Se chemical shift of diselenide precursors, and selenium partial charge in benzeneselenol derivatives – offering predictive power for bandgap tuning.

## Results and Discussion

AgSePh (Figure 1a) is a two-dimensional (2D) van der Waals semiconductor adopting a layered structure similarly to 2D halide perovskites^36,37^ and transition metal dichalcogenides.^38^ In each AgSePh layer, the Ag atoms form a hexagonal sheet that is surrounded above and below the plane by the SePh units. Each AgSePh layer then stacks on top of another via van der Waals interactions forming a three-dimensional (3D) crystal of layered materials.

**Figure 1:**
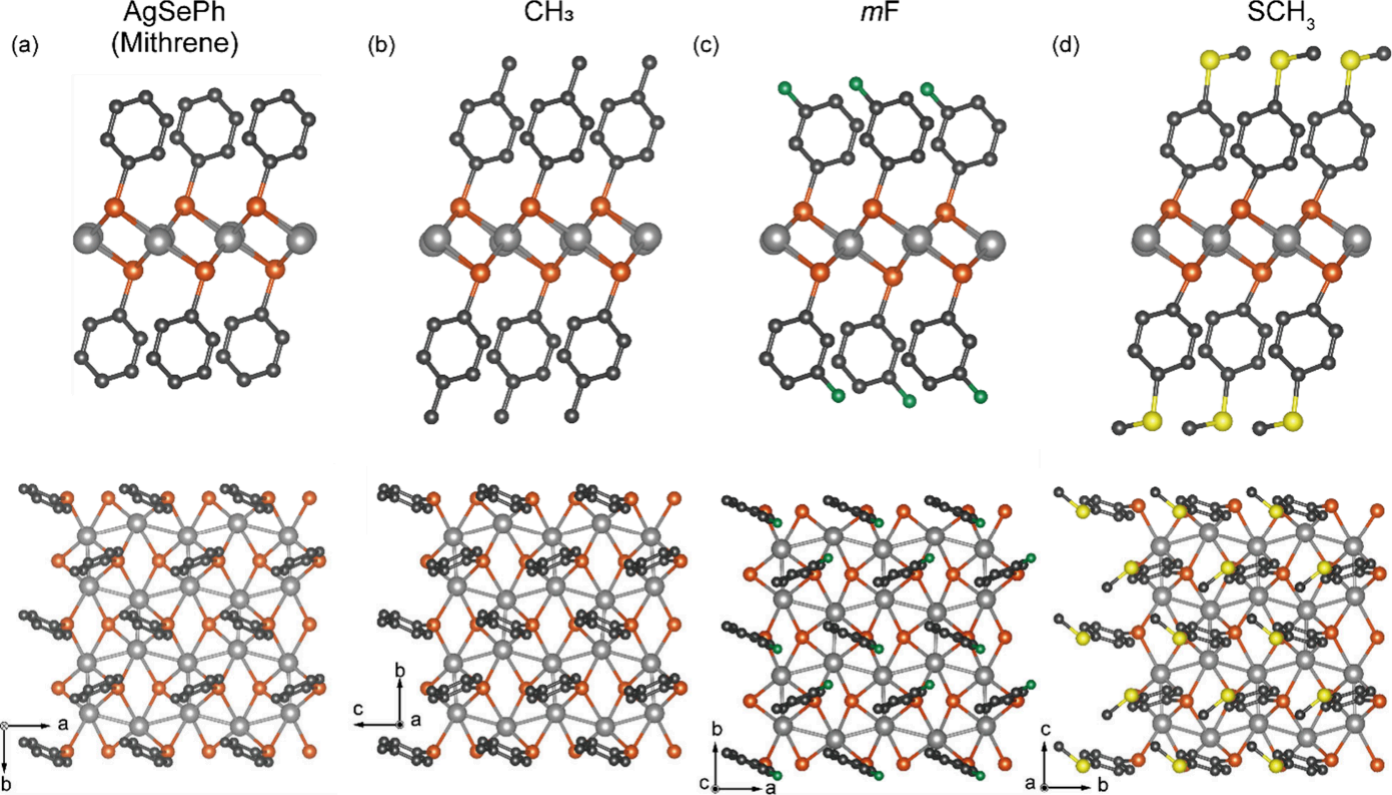
Crystal structures of selected mithrene variants. Crystal structures of (a) AgSePh (mithrene), referred to as **H**, (b) **CH**_**3**_, (c) *m* **F**, and (d) **SCH**_**3**_. Ag, Se, C, F, and S atoms are depicted by gray, orange, black, and green, and yellow spheres, respectively. Hydrogen and disordered atoms are omitted for clarity.

In this study, we synthesized AgSePh (which we will abbreviate as **H**) and eight analogues with modified organic components chosen because of their electron-donating/withdrawing characteristic. These include six members with para-substituted functional groups – **CF**_**3**_, **CH**_**3**_, *p* **F**, **OC**_**8**_ **H**_**17**_, **SCH**_**3**_, and **N­(CH**_**3**_ **)**_**2**_) – one member with a meta-substituted functional group – *m* **F** – and one member with an extended conjugation system – **Napht** (Figure 2a).

**Figure 2:**
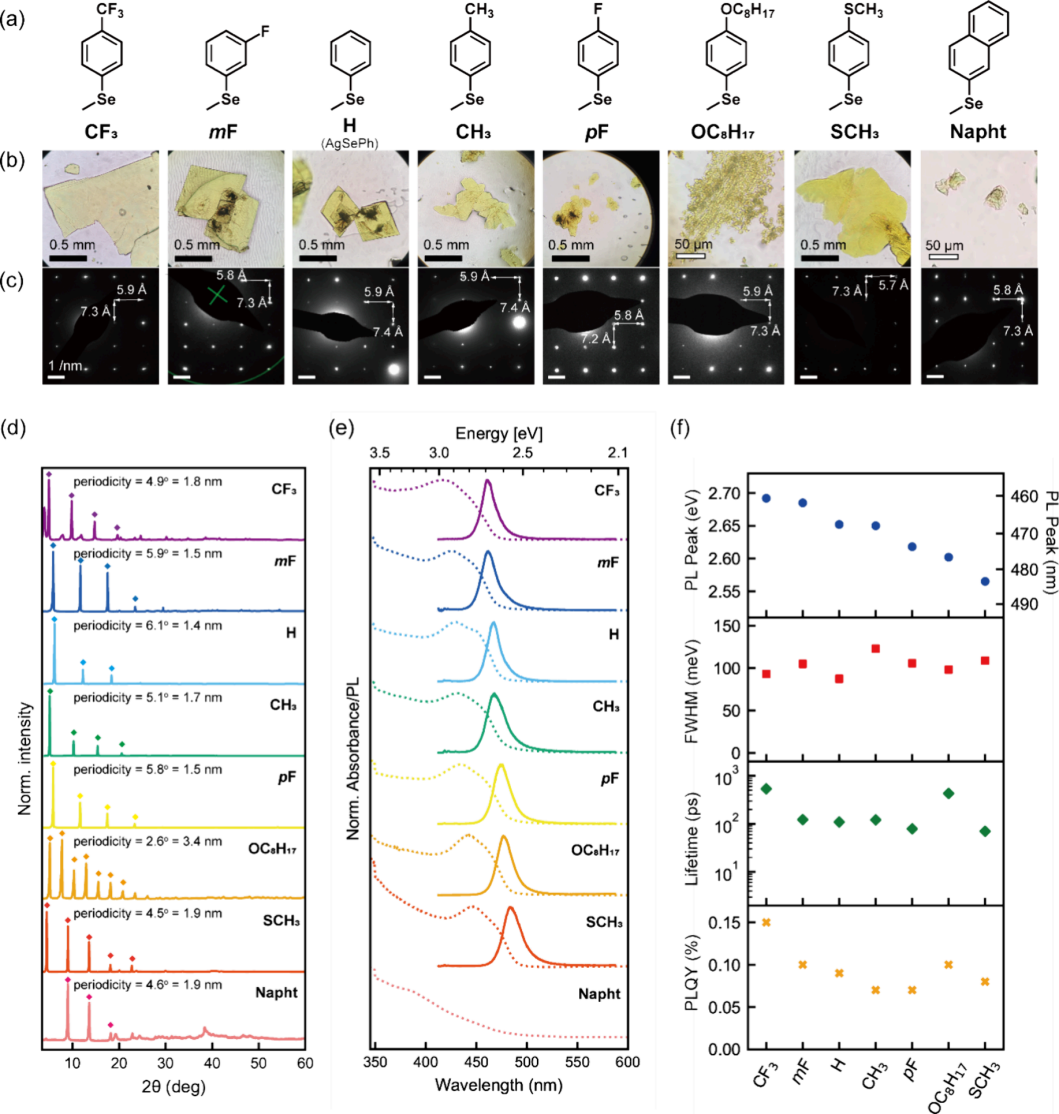
Diffraction and optical properties of synthesized mithrene variants. (a) Molecular structure of selenide ligands. (b) Optical micrographs. (c) Selected area electron diffraction images of mithrene variants. (d) X-ray diffraction patterns of synthesized compounds with diamond symbols indicating periodic diffraction peaks corresponding to 2D structures. (e) Absorption (Abs; dotted line) and photoluminescence (PL; solid line) spectra of the synthesized compounds. (f) PL peak wavelengths, PL full-width-at-half-maximum (fwhm), PL decay lifetime, and PL quantum yield (PLQY) of synthesized compounds.

Mithrene crystals (Figures 2b and S1) were prepared by the amine-assisted crystallization method,^28^ wherein a solution of diphenyl diselenide in toluene is mixed with a solution of silver nitrate (AgNO_3_) in butylamine and left to react at 5 °C for 1–3 days. AgSePh-R variants were prepared similarly using organodiselenide precursors synthesized via Grignard reactions, with water diffusion or addition of triphenyl phosphine as needed to control the reaction rate (see Table S1 and Section 2.2 of the Supporting Information for detailed synthesis conditions). All AgSePh-R variants were found to be stable under ambient storage for several weeks and show no obvious degradation in water at pHs 1, 7 and 14, as well as in isopropanol and toluene solvents (Figure S2).

Using the above synthesis methods, we were able to obtain single crystals and identify the structures of three new members of the MOC family – *m* **F**, **SCH**_**3**__,_ and **N­(CH**_**3**_ **)**_**2**_ – in addition to the previously discovered structures of **H** and **CH**_**3**_ reported in our earlier publication.^28^ The structures of *m* **F**, **SCH**_**3**_, **H**, and **CH**_**3**_ and their crystallographic information are shown in Figure 1 and Table 1. Single-crystal X-ray diffraction (SCXRD) revealed that *m* **F** crystallized in the orthorhombic *P*2_1_2_1_2_1_ space group at 100 K. For **SCH**_**3**_, we were able to resolve its structure through Rietveld refinement of synchrotron X-ray diffraction (XRD) data (Figure S3 and Table S2). Similar to **H** and **CH**_**3**_, *m* **F** and **SCH**_**3**_ exhibit hybrid 2D van der Waals crystals, with covalent bonding between Ag and Se atoms (as inferred from their electronegativity difference of ∼0.6), forming 2D sheets surrounded by organic ligands (Figure 1). In this structure, Se atoms are arranged into layers above and below each Ag layer, and the phenyl (Ph) rings that are covalently bound to the Se atoms are oriented perpendicular to the 2D plane and passivate the surfaces of the inorganic AgSe cores. The Ag atoms form a distorted hexagonal pattern (Figures 1 and S4). Although slight variations in the distance between Ag–Ag or Ag–Se bonds were observed among these MOCs (Table S3), the overall crystal structure remained faithful to mithrene despite the *meta*- and *para*-modifications.

**Table 1:** Crystal Data and Structural Refinement of *m* **F** and Colorless **N­(CH**_**3**_ **)**_**2**_ (1D), **H** and **CH**_**3**_ ^a^

	*m*F	N(CH_3_)_2_ (1D)	H	CH_3_
Empirical formula	C_6_H_4_AgFSe	C_19_H_29_Ag_2_N_3_Se_2_	C_12_H_10_Ag_2_Se_2_	C_14_H_14_Ag_2_Se_2_
*M*_r_	281.92	673.11	527.86	555.91
Temperature (K)	100(2)	100(2)	100(2)	100(2)
Wavelength (Å)	0.71073	0.71073	0.71073	0.71073
Crystal system	Orthorhombic	Triclinic	Monoclinic	Monoclinic
Space group	*P*2_1_2_1_2_1_	*P*-1	*P*2_1_/*c*	*P*2_1_/*c*
*a* (Å)	5.8485(6)	9.5331(11)	5.8334(5)	17.2752(10)
*b* (Å)	7.2540(8)	19.823(2)	7.2866(6)	7.2661(4)
*c* (Å)	30.331(3)	23.524(3)	29.079(3)	5.7676(3)
α (°)	90	100.183(3)	90	90
β (°)	90	91.617(3)	95.5819(16)	99.117
γ (°)	90	101.876(3)	90	90
*V* (Å^3^)	1286.8(2)	4272.1(8)	1230.16(18)	714.82(7)
*Z*	8	8	4	2
Calculated density (Mg/m^3^)	2.910	2.093	2.850	2.583
Absorption coefficient (mm^–1^)	8.699	5.250	9.067	7.809
*F*(000)	1040	2608	976	520
Crystal size (mm)	0.250 × 0.215 × 0.035	0.270 × 0.080 × 0.010	0.230 × 0.220 × 0.020	0.230 × 0.215 × 0.010
θ range for data collection (°)	2.686 to 32.021	2.50 to 30.56	2.815 to 31.540	2.803 to 30.994
Index ranges	–8 *≤ h* ≤ 8, −10 ≤ *k* ≤ 10, −45 ≤ *l* ≤ 45	–13 ≤ *h* ≤ 13, −28 ≤ *k* ≤ 28, −33 ≤ *l* ≤ 33	–8 ≤ *h* ≤ 8, −10 ≤ *k* ≤ 10, −42 ≤ *l* ≤ 42	–25 ≤ *h* ≤ 25, −10 ≤ *k* ≤ 10, −8 ≤ *l* ≤ 8
Reflections collected	36902	308047	37922	31849
Independent reflections	4459 [*R*_*int*_ = 0.0598]	26088 [*R*_*int*_ = 0.0682]	4107 [*R*_int_ = 0.0381]	2277 [*R*_int_ = 0.0365]
Completeness to θ = 25.242°	99.6%	100.0%	99.5%	99.5%
Absorption correction	Semiempirical from equivalents	Semiempirical from equivalents	Semiempirical from equivalents	Semiempirical from equivalents
Refinement method	Full-matrix least-squares on *F*2	Full-matrix least-squares on *F*2	Full-matrix least-squares on *F*2	Full-matrix least-squares on *F*2
Data/restraints/parameters	4459/0/163	26088/42/981	4107/32/145	2277/0/84
Goodness-of-fit on *F*2	1.087	1.032	1.155	1.147
Final *R* indices [*I* > 2σ(*I*)]	*R*1 = 0.0641, *wR*2 = 0.1867	*R*1 = 0.0300, *wR*2 = 0.0606	*R*1 = 0.0341, *wR*2 = 0.1051	*R*1 = 0.0214, *wR*2 = 0.0563
R indices (all data)	*R*1 = 0.0682, *wR*2 = 0.1906	*R*1 = 0.0495, *wR*2 = 0.0669	*R*1 = 0.0393, *wR*2 = 0.1090	*R*1 = 0.0239, *wR*2 = 0.0588
Largest diff. peak and hole (e.Å^–3^)	3.957 and −4.837	1.297 and −1.057	1.817 and −2.419	0.534 and −0.978

a The data for **H** and **CH**_**3**_ are obtained from ref 28.

For **N­(CH**_**3**_ **)**_**2**_, we obtained a mixture of nonemissive, colorless crystals and emissive, yellow crystals (Figure S5). Using SCXRD, we determined that the colorless **N­(CH**_**3**_ **)**_**2**_ crystals adopt a one-dimensional (1D) structure with a chemical formula of [AgSePh-N­(CH_3_)_2_]_2_·PrNH_2_, where 1-propylamine cocrystallizes with 1D AgSePh-N­(CH_3_)_2_ (Figure S6). The 1D chain is composed of a Ag_8_Se_8_ core as a repeating unit, which is surrounded by organic ligands. Unlike previously reported Ag-based 1D MOCs such as AgSePh-F_2_(2,6)^19^ and [Ag­(*o*-SPhCO_2_H)]_n_ ^31^ whose cores are composed of Ag–Ag interactions along the direction of the chain, Ag interactions in **N­(CH**_**3**_ **)**_**2**_ are segmented into Ag_4_ units, which are connected to each other through Se atoms. On the other hand, we were unable to determine the structure of the yellow **N­(CH**_**3**_ **)**_**2**_ sample, but we suspect that it likely adopts a 2D structure due to the similarity of their absorption and PL spectra with other 2D MOCs synthesized in this work (Figure S5c).

Crystals of **CF**_**3**_, *p* **F**, **OC**_**8**_ **H**_**17**_, and **Napht** were not of sufficient quality for X-ray crystallography. However, we employed selected area electron diffraction (SAED) and powder X-ray diffraction (PXRD) to confirm their 2D structure and similarity to those of mithrene. Figure 2c shows SAED patterns collected with the electron beam at normal incidence to the 2D plane, for all synthesized compounds except **N­(CH**_**3**_ **)**_**2**_. The SAED results suggest similar in-plane unit cell parameters for all of the 2D MOCs, with one unit cell parameter ranging from 5.7 to 5.9 Å and the other from 7.2 to 7.4 Å, indicating that these MOCs likely adopt structures similar to those of **H**, **CH**_**3**_, *m* **F** and **SCH**_**3**_. PXRD patterns are displayed in Figure 2d; each compound shows periodic diffraction peaks below 2θ = 20°, consistent with the adoption of 2D van der Waals structures. The spacings of these diffraction peaks, which vary depending on the choice of the organic component, correspond to interlayer separations of 1.44–1.94 nm. Notably, **OC**_**8**_ **H**_**17**_, with its long hydrocarbon chain, shows the largest interlayer separation of 3.4 nm (Table S5).

Thermogravimetric analysis (TGA) revealed that all synthesized MOCs are stable up to at least ∼250 °C (Figure S7). The extracted *T*_d5%_ (temperature at 5% weight loss) values of most MOC samples are between 248 and 258 °C, except for electron-rich **SCH**_**3**_ and **Napht** whose values were higher at 266 and 294 °C, respectively. Analysis of the remaining weights and the PXRD patterns of the TGA residues at 500 °C (Figure S8 and Table S6) show that **CF**_**3**_, *m* **F**, **H**, **CH**_**3**_ and *p* **F** decompose into Ag, agreeing with previous reports.^35,39^ However, the decomposition of MOCs with electron-rich organic units like **OC**_**8**_ **H**_**17**_, **Napht** and **SCH**_**3**_ yields Ag_2_Se or a mixture of Ag and Ag_2_Se as the decomposition product.^40,41^ TGA and PXRD analyses of **CF**_**3**_ showed that the sample may contain an impurity, which vaporizes at ∼150 °C (Figures 2d and S6).

The absorption and PL spectra of the synthesized AgSePh-R compounds, along with other optical properties, are presented in Figures 2e, 2f, S5, and S9. All 2D MOCs, except **Napht**, exhibit narrow-line width PL at room temperature, with PL peak positions tunable from 461 nm (**CF**_**3**_) to 486 nm (yellow **N­(CH**_**3**_ **)**_**2**_ crystals), covering a broad range from blue to turquoise in the Commission Internationale de l’Éclairage (CIE) color space (Figures S10 and S11). We observed that electron-donating ligands, such as **N­(CH**_**3**_ **)**_**2**_, **SCH**_**3**_, and **OC**_**8**_ **H**_**17,**_ induced a red-shift in the PL peak, while electron-withdrawing ligands caused a blue-shift, suggesting that PL peak shifts arise from electronic effects of the organic functional group. In contrast to other 2D MOCs, **Napht** showed weak, broad luminescence centered at 740 nm (Figure S9). This emission peak is longer than both the photoluminescence and the excimer emission of naphthalene,^42^ suggesting that the emission originates from a new electronic state arising from MOC formation.

The narrow full-width-at-half-maximum (fwhm) of mithrene was maintained at 100–150 meV regardless of the added functional groups. Additionally, time-resolved PL analysis showed single-exponential decays for all 2D MOC samples with decay constants of 70–500 ps (Figures 2f and S12), and photoluminescence quantum yield (PLQY) measurement of MOC crystals dispersed in 2-propanol gave the PLQY values of 0.07–0.15% (Figure 2f). Similar PLQY and PL decay lifetimes across all mithrene variants suggest a common nonradiative recombination pathway that has yet to be identified.

To understand the electronic effects of phenyl ring modification on mithrene, we performed electronic structure calculations using DFT on the four MOCs with known crystal structures (*m* **F**, **H**, **CH**_**3**_, and **SCH**_**3**_). Using experimentally obtained structures as the starting points, we began the calculation by relaxing their structures with DFT. Relaxation had minimal impact on the positions of heavy atoms, causing only slight shifts in the H positions, except for **SCH**_**3**_ – see Figure S13. Figure 3 shows the calculated band structures from the DFT-optimized structures. Both *m* **F** and **H** exhibit direct band gaps at Γ points, while **CH**_**3**_ and **SCH**_**3**_ display indirect band gaps, with the conduction band minima (CBM) at Γ points and the valence band maxima (VBM) at X points (Figure S14). Due to the small energy difference between Γ and X points (<3 meV), **CH**_**3**_ and **SCH**_**3**_ can be effectively considered as direct band gap semiconductors.

**Figure 3:**
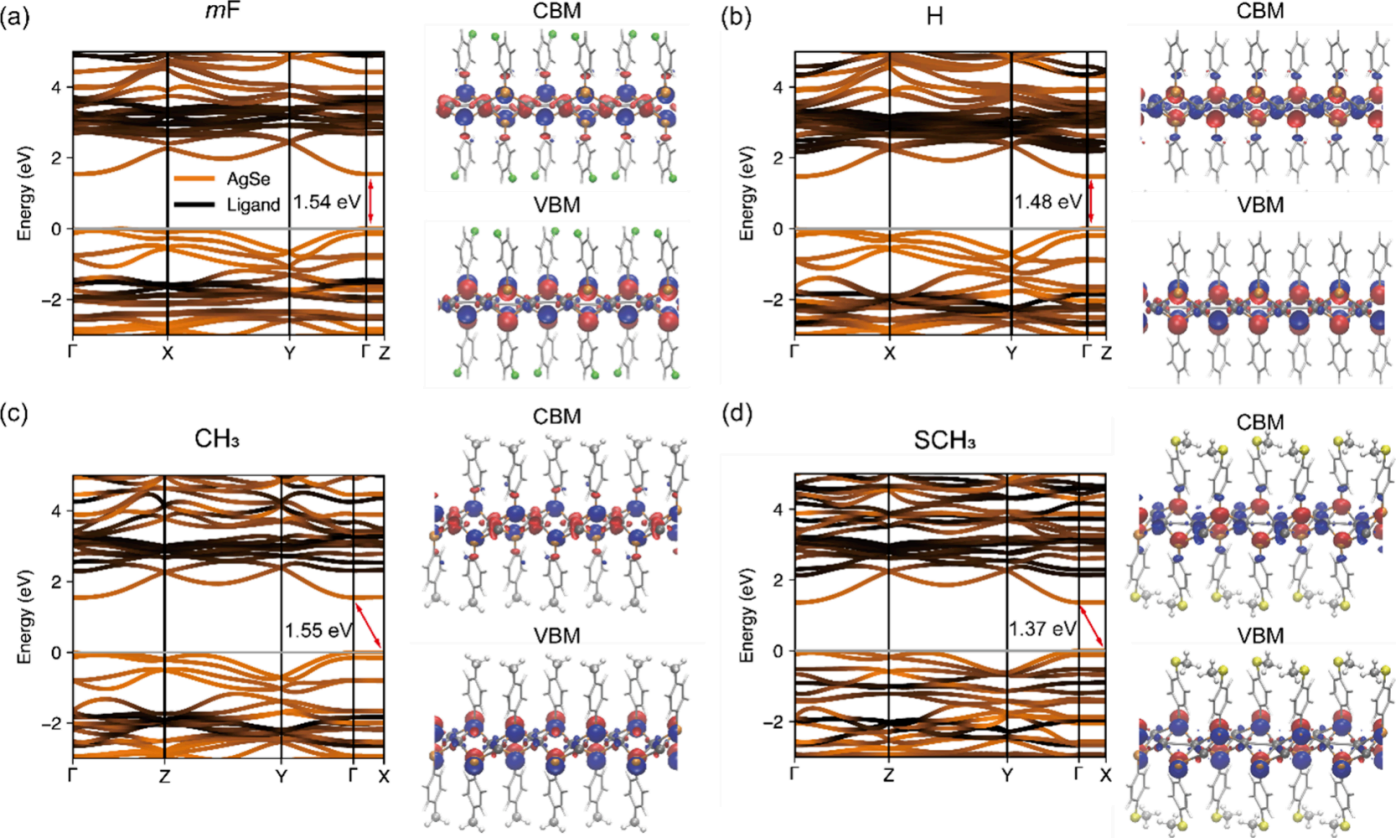
Electronic structure calculation. Electronic band structure and wave functions of the CBM and VBM at the Γ point of (a) *m* **F**, (b) **H**, (c) **CH**_**3**_, (d) **SCH**_**3**_ calculated using DFT with the PBE functional. The Brillouin zones of these four structures are shown in Figure S14. The bands are colored according to the contribution from each component, AgSe in orange and ligand in black. Blue and red surfaces represent the positive and negative phases of the wave function, respectively. The isosurface value is set at 0.002 e/Bohr^3^. Atoms are as follows: Ag: dark gray, Se: orange, C: light gray, F: green, S: yellow, H: white. The Ag and Se elements as well as added functional groups are shown as spheres, while the remaining atoms are shown as sticks.

The band gaps of *m* **F**, **H**, and **CH**_**3**_ are particularly close with calculated values of 1.54, 1.48, and 1.55 eV, respectively – a difference of no more than 0.07 eV. While the ordering of the band gaps does not directly match the ordering of the PL peak wavelengths, the small difference in band gaps falls within the typical margin of DFT calculation error. These three MOCs show ligand contributions of around 2 eV below the VBM and 1.5 eV above the CBM. **SCH**_**3**_, on the other hand, has a notably lower DFT-computed band gap than the others. Additionally, **SCH**_**3**_ features a distinctive band composition, with the S contribution positioned around 0.8 eV below the VBM (Figure S15). A closer energetic alignment of the **SCH**_**3**_ ligand contribution to the VBM results in a distinct shape of the lower valence bands near the VBM compared to those of the other three MOCs.

The influence of the inorganic framework distortion and the chemical nature of the functional group was investigated separately by calculating the direct band gap while varying one component at a time (Figure S16). When the functional group is changed from **H** to either *m* **F** or **CH**_**3**_ while the inorganic framework is kept unchanged, the direct band gap decreases by 0.05 eV. In contrast, taking the distorted inorganic framework (e.g., from *m* **F** or **CH**_**3**_) and combining with the phenyl ring alone, the direct band gap increases to within 0.01 eV of the fully functionalized counterparts. For the fixed framework, **SCH**_**3**_ functionalization decreases the direct band gap by 0.05 eV, but a larger 0.08 eV decrease is observed when the inorganic framework is distorted, while the phenyl ring remains unfunctionalized. These results indicate that distortion of the inorganic framework induced by functionalization contributes significantly to changes in the band gap. However, unambiguously assigning these structural distortions to electronic induction effects vs steric interactions among the ligands is challenging.

The frontier orbitals at the CBM and VBM are depicted alongside the electronic band structures in Figure 3. Overall, we found the four MOCs to exhibit a similar density of states projected onto each atomic orbital (Figure S15). Electron density at the VBM is localized solely on the Ag and Se atoms. The probability density distribution of electrons at the CBM is similarly found on the AgSe core but can also be found on the adjacent C atoms.

Considering that the frontier orbitals at the CBM and VBM have significant contributions from Ag and Se atoms, we hypothesized that organic modification could modify the band gaps by pushing and pulling electron density on the AgSe cores. As changes to semiconductors’ electronic band gaps can be revealed through shifts in PL arising from recombination of charge carriers (a wider band gap leads to blue-shifted emission whereas a reduction in the band gap leads to red-shifted emission), we plotted the emission wavelengths of AgSePh-R variants and found correlation with three parameters specific to the organic ligand: (i) the Hammett substituent constant of functionalized groups, σ_R_, (ii) ^77^Se chemical shift in nuclear magnetic resonance (NMR) analysis of the organic diselenide precursors, and (iii) the partial charge on Se of a benzeneselenol with corresponding functional groups estimated using natural population analysis of DFT electron densities.^43^ The Hammett constant is an empirical quantity that has been successfully used to predict the influence of functional groups on chemical equilibrium and reaction kinetics.^44^ The ^77^Se chemical shift and the partial charge of Se give information on the electron density around the Se atoms.^45^ All of these parameters quantifies the electron-withdrawing or electron-donating nature of a substituent group.^46^ Figure 4a summarizes the PL spectra of the synthesized MOCs. In general, we found that increasing the PL peak wavelength is accompanied by decreasing the Hammett substituent constant (Figure 4b), increasing the ^77^Se NMR chemical shift (Figure 4c), and decreasing the partial charge of Se (Figure 4d), suggesting that the electron-donating or electron-withdrawing character of the ligand is the driving force for PL peak wavelength tuning.

**Figure 4:**
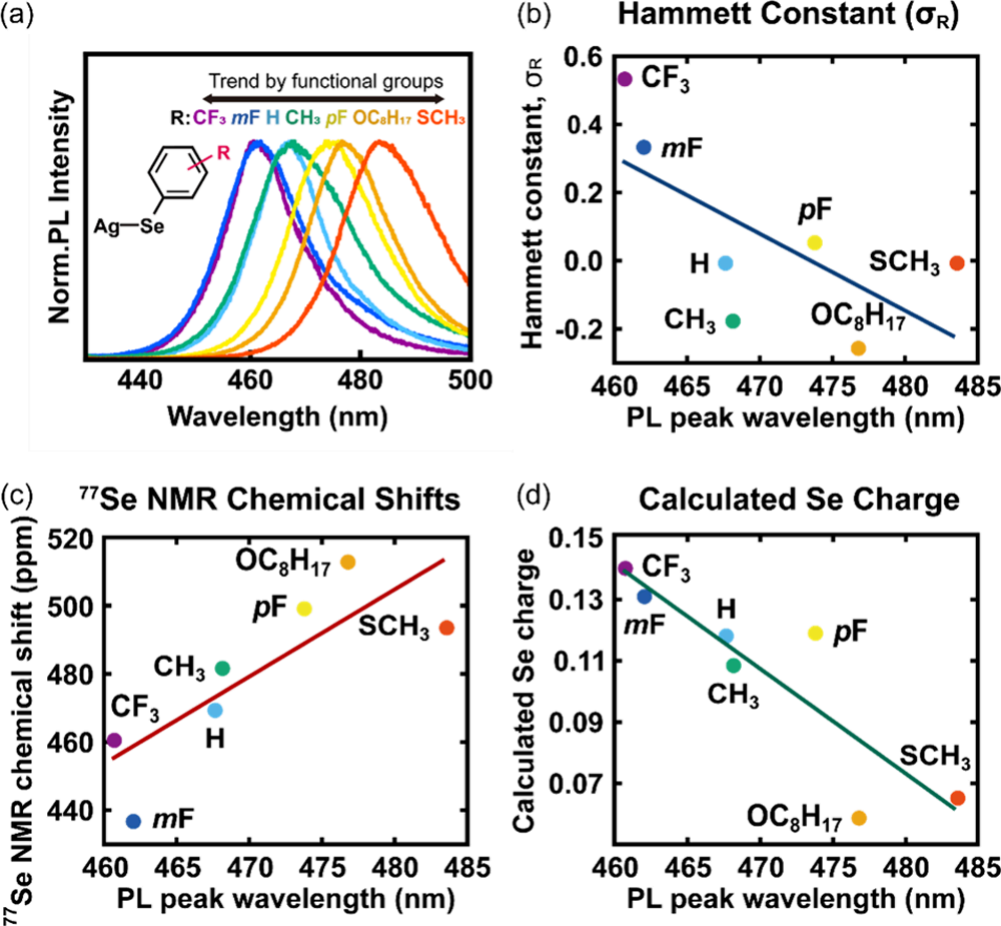
Correlations between AgSePh-R emission peak and ligand charge parameters. (a) PL peak shifts of synthesized MOCs. (b) Correlation between the Hammett constant and PL peak wavelength. (c) Correlation between the ^77^Se chemical shift value of diselenide precursors and PL peak wavelength. (d) Correlation between the calculated Se partial charge of benzeneselenols with corresponding functional groups and PL peak wavelength.

## Conclusion

In summary, we demonstrate systematic optical band gap tuning via organic ligand engineering of silver based MOCs. A total of nine MOCs, including five novel compounds, were synthesized using electron-donating, electron-withdrawing, or long-conjugated ligands. All MOCs exhibit 2D van der Waals crystals similarly to AgSePh, except **N­(CH**_**3**_ **)**_**2**_ that cocrystallized with PrNH_2_ to form a 1D structure. As the electron-donating ability of the functional groups increased, the PL spectra red-shifted from 2.53 to 2.69 eV, allowing for coverage from blue to turquoise in the color space. DFT calculations revealed that although the frontier orbitals are mostly located within the inorganic component, the functional group in the organic component affects the electron density around the Se atoms, allowing for precise tuning of the optical gaps. Additionally, we found three parameters – the Hammett constant of functional groups, ^77^Se chemical shifts of diselenide precursors, and the partial charge of Se of a benzeneselenol with a corresponding functional group – as good predictive parameters to estimate the shifts in PL positions.

Overall, these observations demonstrate that by obtaining the Hammett constant, ^77^Se NMR chemical shift, or partial charge of Se of the organic components with various substitutions, the PL peak emission wavelength of the substituted mithrene compounds can be estimated. Hence, these three parameters, especially the partial charge of Se which can be calculated beforehand, can serve as predictive tools for guiding the design of MOCs. Additionally, the observed correlations underscore the true hybrid nature of MOCs, where the organic component directly influences the inorganic component and the frontier orbitals to enable more precise control over the band gap. These results suggest MOCs behave as truly hybrid semiconductors and provide effective design parameters for controlling their properties. This work lays important foundation in property tuning of MOCs and facilitate future studies of this emerging family of covalent-bonded hybrid semiconductors.

## Supplementary Material



## Notes

Experimental details, synthesis and characterization of organic precursors, additional structural and characterization data for synthesized MOCs, and theoretical calculation results (PDF)
