# Supplementary material for: Systematic Bandgap Engineering of a 2D Organic–Inorganic Chalcogenide Semiconductor via Ligand Modification
Source: J Am Chem Soc. 2025 Aug 19;147(35):31704–12. doi: 10.1021/jacs.5c07989 (PMC12412171; doi:10.1021/jacs.5c07989)
Supplement: Supplementary file 1 [file ja5c07989_si_001.pdf]

Supporting information for

Systematic Bandgap Engineering of a 2D Organic-Inorganic Chalcogenide Semiconductor *via* Ligand Modification

Tomoaki Sakurada,[1‡] Watcharaphol Paritmongkol,[1,2‡]* Yeongsu Cho,[1] Woo Seok Lee,[1,3] Petcharaphorn Chatsiri,[2,4] Julius J. Oppenheim,[5] Ruomeng Wan,[1,5] Annlin Su,[1] Nicholas Samulewicz,[1] Khemika Wannakan,[2] Peter Müller,[5] Mircea Dincă,[5] Heather J. Kulik,[1,5] William A. Tisdale[1]*

[1]Department of Chemical Engineering, Massachusetts Institute of Technology, Cambridge, Massachusetts 02139, United States

[2]Department of Materials Science and Engineering, School of Molecular Science and Engineering, Vidyasirimedhi Institute of Science and Technology (VISTEC), Rayong 21210, Thailand

[3]Department of Materials Science and Engineering, Massachusetts Institute of Technology, Cambridge, Massachusetts 02139, United States

[4]Department of Chemical and Biomolecular Engineering, School of Energy Science and Engineering, Vidyasirimedhi Institute of Science and Technology (VISTEC), Rayong 21210, Thailand

[5]Department of Chemistry, Massachusetts Institute of Technology, Cambridge, Massachusetts 02139, United States

[‡]These authors contributed equally

*Correspondence to: tisdale@mit.edu, watcharaphol.p@vistec.ac.th

Table of Contents

1. Materials and Methods 2
2. Synthesis 6
3. Supplementally Figures and Tables 16
4. References 42

1. Materials and Methods

1.1. Chemicals

Solvents and reagents were purchased from TCI America, Millipore Sigma, or Fisher Scientific and used without any further purification.

1.2. Characterization

Nuclear magnetic resonance (NMR) measurement

NMR spectra were recorded with a Bruker Advance 400 MHz spectrometer at 298 K. The spectral data are reported as chemical shift (in ppm). Chemical shifts were calibrated against peaks of reference chemicals as internal standard (^{1}H-NMR: Tetramethylsilane δ = 0 ppm, ^{19}F-NMR: Trichlorofluoromethane, δ = 0 ppm, ^{77}Se-NMR: Dimethylselenide δ = 0 ppm) or a solvent (^{13}C-NMR: Chloroform δ = 77.16 ppm).

High resolution mass spectroscopy (HRMS)

HRMS was collected using a Jeol AccuTOFDART™ mass spectrometer using DART source ionization.

Thermogravimetric analysis (TGA)

TGA data was collected with a TGA Q500 differential thermal analyzer. The samples were heated from room temperature to 500 °C with a heating rate of 2 °C min^{-1} under N_2 stream (45 mL min^{-1}).

Powder X-ray Diffraction (PXRD)

Powder X-ray diffraction data were collected using a PANalytical X'Pert Pro MPD X-ray diffractometer (Cu K_α radiation, λ = 1.54184 Å) with high-speed Bragg−Brentano optics. A 0.04 rad Soller slit, a 2° anti-scatter slit, a 10 mm mask, and a programmable divergence slit with an illuminated length of 6 mm were used in the incident beam path. The diffracted beam optics included a 0.04 rad Soller slit, a Ni filter, and an automatic receiving slit. The detector was an ultrafast X'Celerator RTMS detector.

UV-Vis Diffuse Reflectance Spectroscopy

Measurements of diffuse reflectance were performed on a Cary 5000 UV-vis-NIR spectrometer equipped with a PIKE Technologies DiffusIR accessory. Solid samples were prepared by grinding with dry potassium bromide (KBr) to a ∼1 wt% dilution and diffuse reflectance spectra were normalized to a 100% KBr baseline. The obtained diffuse reflectance spectra were converted into absorption spectra by Kubelka−Munk transform[1]:

$$F(R) = \frac{(1-R)^2}{2R}$$

where F(R) is the Kubelka−Munk function with a value proportional to the sample's absorption coefficient, and R is the relative reflectance of the sample with the 100% KBr baseline.

Single-Crystal X-ray Diffraction

Low-temperature diffraction data were collected on Bruker-AXS X8 Kappa Duo diffractometers with *IμS* micro-sources using Mo K_{α} radiation (λ = 0.71073 Å), coupled to an APEX2 CCD detector for the structure of X8_21005 (**$N(CH_3)_2$**) and to a Photon 3 CPAD detector for the structure of P8_22014 (***m*F**), performing ϕ-and ω-scans. The structures were solved by dual-space methods using SHELXT[2] and refined against F^2 on all data by full-matrix least squares with SHELXL-2017[3] following established refinement strategies.[4] All non-hydrogen atoms were refined anisotropically. All hydrogen atoms were included into the model at geometrically calculated positions and refined using a riding model. The isotropic displacement parameters, U_{iso}, of all hydrogen atoms were fixed to 1.2 times the U_{eq}-value of the atoms they are linked to (1.5 for methyl groups). Coordinates for nitrogen-bound hydrogen atoms in the structure of **$N(CH_3)_2$** were taken from the difference Fourier synthesis and those atoms were subsequently refined semi-freely with the help hf N—H distance restraints (target value 0.91(2) Å while constraining their U_{iso} as described above.

Compound **$N(CH_3)_2$** crystallizes in the triclinic centrosymmetric space group *P*-1 with eight monomeric subunits and two molecules of *n*-propylamine in the asymmetric unit.

Compound ***m*F** crystallizes in the orthorhombic chiral centrosymmetric space group $P2_12_12_1$ with two monomeric subunits in the asymmetric unit. The quality of the crystal was low. Significant positive and negative residual electron density located near the metal atoms is probably an artefact of packing problems (the diffraction pattern shows relatively high diffuse scattering and streaks). Interpretation of one of the high residual density maxima (namely Q3) as bridging oxygen was attempted and did not result in a sensible model. The other residual peaks are too close to metal atoms to be anything but spurious. Non-merohedral twinning was ruled out and a model based on whole-molecule disorder could not be established.

Synchrotron X-ray Diffraction

High-resolution synchrotron powder X-ray diffraction for AgSePh-SCH_3 was performed using the mail-in program at beamline 11-BM at the Argonne National Laboratory (ANL) Advanced Photon Source (APS). Samples were packed into a Kapton capillary and the ends were sealed using epoxy. Samples were mounted into Kapton capillary sample holders provided by the beamline and shipped to the beamline. Powder patterns were collected at 100 K using 0.458975 Å X-rays.

The refinement was performed as a Rietveld refinement with energies in BIOVIA Materials Studio 2020 using the Reflex module. The weighting scheme was set at 95% Rietveld and 5% force field. The Rietveld was performed over the range of 0.5° to 18°. The peak shape was refined as a Pearson VII function. The force field was the Universal force field using QEq charges.

Selected Area Electron Diffraction

Selected Area Electron Diffraction data were obtained with a Talos Arctica G2 transmission electron microscope with a Ceta 16M Camera. The accelerating voltage is set at 200 kV. Samples were prepared by sonicating the MOCs (~2 mg) in methanol (~2 mL) for 60s. After the sample reaches a colloidal dispersion state, the MOCs dispersion is drop-casted onto C-flat Cu TEM grids with holey carbon film. Prior to electron diffraction data collection, TEM images were obtained to identify regions with uniform crystalline morphology that are suitable for selected area electron diffraction data collection. The collection dose rate was set at 35.60 el/Å^2.

Photoluminescence (PL) spectrum

Steady-state PL measurements were performed on an inverted microscope (Nikon, Ti-U Eclipse) in air. The samples were excited by focusing the output of a 405 nm laser diode (Picoquant, LDHDC405M, continuous-wave mode) by an objective lens (Nikon, CFI S Plan Fluor ELWD, 40×, 0.6 NA) to ~1 μm spot. The polarization of the excitation was controlled to be circularly polarized with a circular polarizer (Thorlabs, CP1R405). After the excitation, the PL was collected in the epi configuration, and passed through a dichroic mirror and a long-pass filter. Then, it was directed into a spectrograph (Princeton Instruments, SP-2500) mounted with a cooled charge-coupled detector (Princeton Instruments, Pixis).

Time-Resolved PL Spectroscopy

Time-resolved PL measurements were performed using the same microscope setup as steady-state PL spectroscopy with some modifications. A variable repetition-rate 405 nm pulsed laser diode (Picoquant, LDHDC-405M) was used as the excitation light source. The detector used was a Si avalanche photodiode (Micro Photon Devices) connected to a counting board for time-correlated single-photon counting (PicoQuant, PicoHarp 300).

Photoluminescent Quantum Yield.

The measurement of PL quantum yield (QY) was performed at room temperature using the absolute quantum yield method in an integrating sphere.[5] A colloidal dispersion of MOCs was prepared by sonication of crystals of MOCs in 2-propanol (*ca.* 0.5 wt%) and then placed into a quartz cuvette with a 10 mm path length. The excitation light from a 405 nm laser diode (Picoquant, LDHDC-405M, continuous wave mode) was directed into an integrating sphere (Labsphere) containing the sample. The output signal was collected by an optical fiber mounted at an exit port of the integrating sphere and directed to a fiber-optic spectrometer (Avantes).

1.3. Density functional theory (DFT) calculations

Electronic structure calculations

DFT calculations of the MOCs were performed using Vienna Ab initio Simulation Package (VASP) version 6.3.1.[6–9] The PBE functional[10] was used in combination with projector augmented wave pseudopotentials[11] with a kinetic energy cutoff of 1000 eV. Dispersion corrections were applied using the empirical DFT-D3[12] with Becke-Johnson damping.[13] Atomic positions were optimized with fixed cell parameters, starting from the crystal structure obtained by PXRD. A 3×3×1 k-mesh was used, and optimization continued until the total energy difference between steps fell below 1 meV. Self-consistent field calculations were performed on the optimized geometry using a 6×6×1 k-mesh to obtain the projected density of states and the charge density. The band structure was then calculated based on the charge density obtained with this higher k-point mesh density.

Partial charge of Se

DFT calculations of isolated benzeneselenols with corresponding functional groups were performed using ORCA version 5.0.0.[14] The B3LYP functional[15–17] and def2-TZVP basis set[18] were applied. To expedite calculations, the RIJCOSX approximation was used, along with def2/J[19] and def2-TZVP/C[20] auxiliary basis sets. Dispersion interactions were included by applying empirical D3[12] corrections with Becke-Johnson damping.[13] The geometry of benzeneselenol was first optimized, followed by natural population analysis[21] to determine the natural charge as the partial charge.

2. Synthesis

2.1 Synthesis of diselenides

General procedure for synthesis of diselenides[22]

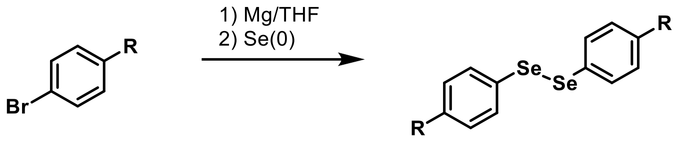

To a dispersion of Mg (0.8 g, 33 mmol) and 20 mL of anhydrous tetrahydrofuran (THF), a small amount of arylbromide (5 mmol) was added and stirred under N_2 atmosphere for 20 min. Once the internal temperature increased, a second portion of arylbromide (25 mmol) in THF (10 mL) was added dropwise to a vigorously stirred dispersion over 15 min. The reaction mixture was heated to 50 °C and stirred for 1 h before being cooled in an ice bath. Elemental selenium (2.6 g, 33 mmol) was added in a single portion, and the reaction mixture was allowed to warm to room temperature and stirred for 1 h. The mixture was then filtered thorough Celite and left at ambient condition overnight. After that, the solvent was evaporated, and the obtained residue was redissolved in dichloromethane. This organic phase was washed sequentially with an aqueous ammonium chloride solution and brine, then dried over Na_2SO_4. Solvent was evaporated again under reduced pressure using a rotary evaporator, and the crude product was purified by column chromatography (using hexane as the eluent). Diselenides containing naphtyl, thiomethyl, and methyl group were reprecipitated from dichloromethane and hexane. Other diselenides were initially obtained as oils, which later transformed into yellow or orange crystals when stored at ambient conditions for 1-2 weeks. For the diselenides which have already been reported, the synthesis was verified by cross-referencing the experimental data with the results reported in the previously published studies.

1,2-Bis(4-trifluoromethylphenyl) diselenide [23]

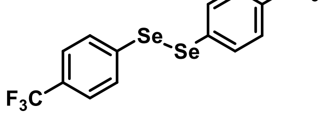

Yellow crystals (3.5 g, 26% yield)

^{1}H NMR (400 MHz, $CDCl_3$) δ 7.72 (d, J = 8.2 Hz, 4H), 7.53 (d, J = 8.2 Hz, 4H). ^{13}C NMR (101 MHz, $CDCl_3$) δ 134.9, 130.6, 129.9 (q, J = 32 Hz, C-CF_3), 126.1, 123.9 (q, J = 273 Hz, C-F_3). ^{19}F NMR (376 MHz, $CDCl_3$) δ –63.1. ^{77}Se NMR (101 MHz, $CDCl_3$) δ 461.2

HRMS calculated for $C_{14}H_8F_6Se_2$ $[M]^+$ 449.8855, found 449.8864.

1,2-Bis(3-fluorophenyl) diselenide

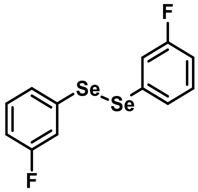

Yellow crystals (2.5 g, 24% yield)

^{1}H NMR (400 MHz, $CDCl_3$) *δ* 7.32 – 7.22 (m, 4H), 7.20 – 7.16 (m, 2H), 6.92 – 6.87 (m, 2H). ^{13}C NMR (101 MHz, $CDCl_3$) *δ* 162.7 (d, *J* = 249 Hz, *C*-F), 132.0 (d, *J* = 6 Hz), 130.5 (d, *J* = 8 Hz) , 126.5 (d, *J* = 3 Hz), 118.1 (d, *J* = 24 Hz), 114.9 (d, *J* = 21 Hz). ^{19}F NMR (376 MHz, $CDCl_3$) *δ* – 114.7. ^{77}Se NMR (101 MHz, $CDCl_3$) *δ* 437.4

HRMS calculated for $C_{12}H_8F_2Se_2$ $[M]^+$ 349.8919, found 349.8938.

1,2-Bis(4-methylphenyl) diselenide [24]

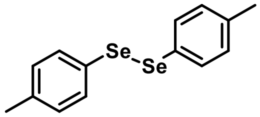

Orange crystals (6.1 g, 60% yield)

^{1}H NMR (400 MHz, $CDCl_3$) *δ* 7.48 (d, *J* = 8.2 Hz, 4H), 7.06 (d, *J* = 7.9 Hz, 4H), 2.33 (s, 6H). ^{13}C NMR (101 MHz, $CDCl_3$) *δ* 137.9, 132.3, 129.9, 127.6, 21.0 (-*C*H_3). ^{77}Se NMR (101 MHz, $CDCl_3$) *δ* 482.3

HRMS calculated for $C_{14}H_{14}Se_2$ $[M]^+$ 341.9421, found 341.9433.

1,2-Bis(4-fluorophenyl) diselenide [24]

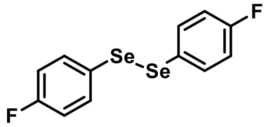

Yellow crystals (3.5 g, 33% yield)

^{1}H NMR (400 MHz, $CDCl_3$) *δ* 7.56 – 7.53 (m, 4H), 6.99 – 6.95 (m, 4H). ^{13}C NMR (101 MHz, $CDCl_3$) *δ* 162.9 (d, *J* = 247 Hz, *C*-F), 134.8 (d, *J* = 8 Hz), 125.6 (d, *J* = 4 Hz), 116.3 (d, *J* = 22 Hz). ^{19}F NMR (376 MHz, $CDCl^3$) *δ* –113.6. ^{77}Se NMR (101 MHz, $CDCl_3$) *δ* 499.8

HRMS calculated for $C_{12}H_8F_2Se_2$ $[M]^+$ 349.8919, found 349.8938.

1,2-Bis(4-octoxyphenyl) diselenide

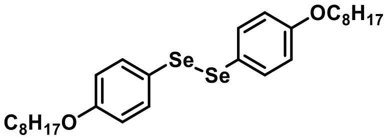

Orange crystals (1.7 g, 10% yield)

^{1}H NMR (400 MHz, $CDCl_3$) δ 7.48 (d, J = 8.8 Hz, 4H), 6.79 (d, J = 8.8 Hz, 4H), 3.95 (t, J = 6.6 Hz, 4H, -OCH_2-), 1.77 (m, 4H), 1.44 (td, J = 9.3, 8.3, 4.4 Hz, 4H), 1.39 – 1.25 (m, 16H), 0.93 – 0.85 (m, 6H, -CH_3). ^{13}C NMR (101 MHz, $CDCl_3$) δ 159.6, 135.5, 121.7, 115.3, 68.1 (O-$C$$H_2$), 31.8, 29.3, 29.2, 26.0, 22.6, 14.0 (-C-H_3). ^{77}Se NMR (101 MHz, $CDCl_3$) δ 513.5

HRMS calculated for $C_{28}H_{42}O_2Se_2$ $[M]^+$ 570.1506, found 570.1510.

1,2-Bis(4-thiomethylphenyl) diselenide

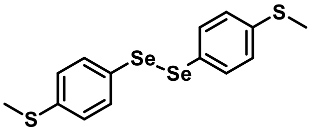

Yellow crystals (7.5 g, 61% yield)

^{1}H NMR (400 MHz, $CDCl_3$) δ 7.50 (d, J = 8.4 Hz, 4H), 7.13 (d, J = 8.4 Hz, 4H), 2.47 (s, 6H). ^{13}C NMR (101 MHz, $CDCl_3$) δ 139.1, 133.0, 127.1, 126.8, 15.4 (S-C-H_3). ^{77}Se NMR (101 MHz, $CDCl_3$) δ 494.72

HRMS calculated for $C_{14}H_{14}S_2Se_2$ $[M]^+$ 405.8862, found 405.8872.

1,2-Bis(2-naphtyl) diselenide [25]

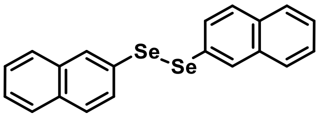

Yellow crystals (6.0 g, 48% yield)

^{1}H NMR (400 MHz, $CDCl_3$) δ 8.08 (s, 2H), 7.83-7.77(m, 2H), 7.76-7.73(m, 2H), 7.73-7.68 (m, 4H), 7.51 – 7.42 (m, 4H). ^{13}C NMR (101 MHz, $CDCl_3$) δ 133.6, 132.7, 130.8, 129.3, 128.8, 128.2, 127.7, 127.5, 126.6, 126.4. ^{77}Se NMR (101 MHz, $CDCl_3$) δ 481.5

HRMS calculated for $C_{20}H_{14}Se_2$ $[M]^+$ 413.9421, found 413.9441.

2.2 Synthesis of MOC crystals

MOC Crystals (**Figures 2b and S1**) were prepared by methods based on amine-assisted crystallization[26] using a mixture of silver nitrate ($AgNO_3$) and organodiselenide precursors that were synthesized *via* Grignard reactions. **Table S1** details the optimized synthesis conditions employed to obtain the crystals. For some organodiselenides with electron-withdrawing groups (**CF_3** and ***m*F**), we observed no crystallization or precipitation from unmodified amine-assisted crystallization but were able to obtain crystals after slow diffusion of water into the $AgNO_3$/organodiselenide solution mixture. For some organodiselenides containing electron-donating groups (**OC_8H_{17}**, **SCH_3**, **Napht,** and **$N(CH_3)_2$**), we observed MOC powders forming immediately after mixing $AgNO_3$ with the organodiselenides. To obtain these crystals, we added a small amount of triphenylphosphine (TPP) to reduce the reaction kinetics. For **$N(CH_3)_2$**) in particular, we obtained a mixture of pale-yellow blade crystals and yellow plate crystals using this synthesis method (**Figure S2**).

*Synthesis of **CF_3** and **mF***

A solution of organodiselenides in toluene (20 mM, 5 mL) was mixed with a solution of $AgNO_3$ in 1-butylamine (20 mM, 5 mL) and filtered through a 0.2 µm PTFE filter. The filtered solution (10 mL) was transferred to a 20 mL glass vial, which was then placed inside a larger 110 mL glass jar containing 20 mL deionized water. This setup allowed water to diffuse into the smaller vial while organic solvents gradually evaporated. The deionized water in the larger glass jar was renewed every 3 days. Crystals began to appear after approximately 6 days at room temperature.

*Synthesis of **CH_3**, **H**, and **pF***

Crystals of **CH_3**, **AgSePh**, or ***p*F** were grown by mixing a solution of diselenides in toluene (10 mM, 10 mL) with a solution of $AgNO_3$ in 1-butylamine (10 mM, 10 mL) at 5 °C for one day.

*Synthesis of **OC_8H_{17}***

Crystals of **OC_8H_{17}** were grown by mixing a solution of 1,2-bis(4-octoxyphenyl) diselenide in toluene (1 mM, 10 mL) with a solution of $AgNO_3$/triphenylphosphine (TPP) in 1-butylamine (1 mM/5 mM, 10 mL) at 5 °C for one night.

*Synthesis of **SCH_3***

Crystals of **SCH_3** were grown by mixing a solution of 1,2-bis(thiomethylphenyl) diselenide in 1-butylamine (1 mM, 5 mL) with a solution of $AgNO_3$/TPP in 1-butylamine (1 mM/10 mM, 5 mL) at 5 °C for three days.

Synthesis of ***Napht***

Crystals of **Napht** were grown by mixing a solution of 1,2-bis(2-naphtyl) diselenide in toluene (1 mM, 10 mL) with a solution of $AgNO_3$/TPP in 1-butylamine (1 mM/5 mM, 10 mL) at 5 °C for one night.

Synthesis of AgSePh-N(CH$_3$)$_2$

Crystals of **N(CH$_3$)$_2$** were grown by mixing a solution of 1,2-bis(dimethylaminophenyl) diselenide in 1-propylamine (1 mM, 10 mL) with a solution of $AgNO_3$/TPP in 1-propylamine (1 mM/5 mM, 10 mL) at 5 °C. Yellow plate crystals and pale-yellow blade crystals began to appear after one night.

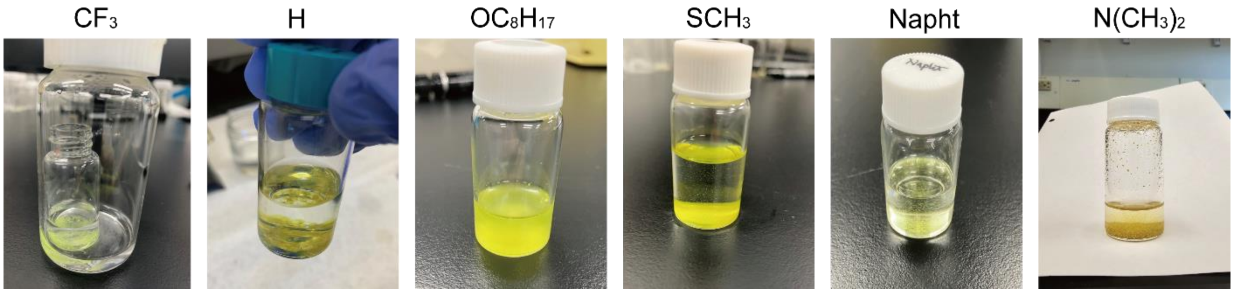

Figure S1. Optical images of reaction solutions after crystals formed.

Table S1. Optimized synthesis conditions for AgSePh-R crystals.

R=	Ag^+			Diselenide		Remark
	$AgNO_3$ [mM]	TPP [mM]	Solvent	Conc. [mM]	Solvent	
**CF$_3$**	20	-	$BuNH_2$	20	Toluene	H_2O diffusion
***m*F**	20	-	$BuNH_2$	20	Toluene	H_2O diffusion
**H**	10	-	$BuNH_2$	10	Toluene	
**CH$_3$**	10	-	$BuNH_2$	10	Toluene	
***p*F**	10	-	$BuNH_2$	10	Toluene	
**OC$_8$H$_{17}$**	1	5	$BuNH_2$	1	Toluene	
**SCH$_3$**	1	10	$BuNH_2$	1	$BuNH_2$	
**Napht**	1	5	$BuNH_2$	1	Toluene	
**N(CH$_3$)$_2$**	1	5	$PrNH_2$	1	$PrNH_2$	Mixture of crystals

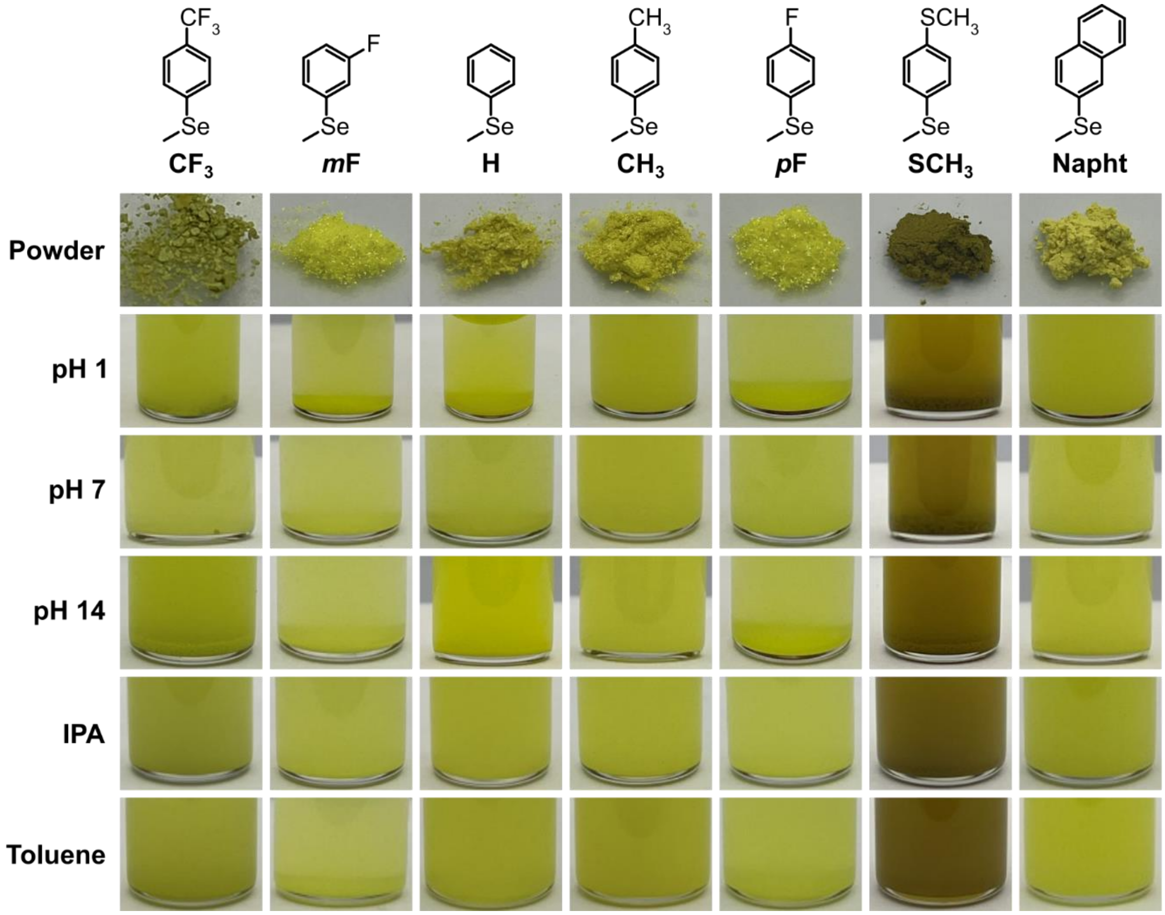

Figure S2. Images of MOC materials under ambient storage in powder forms and storage in water with pHs 1, 7, and 14, as well as in isopropanol (IPA) and toluene.

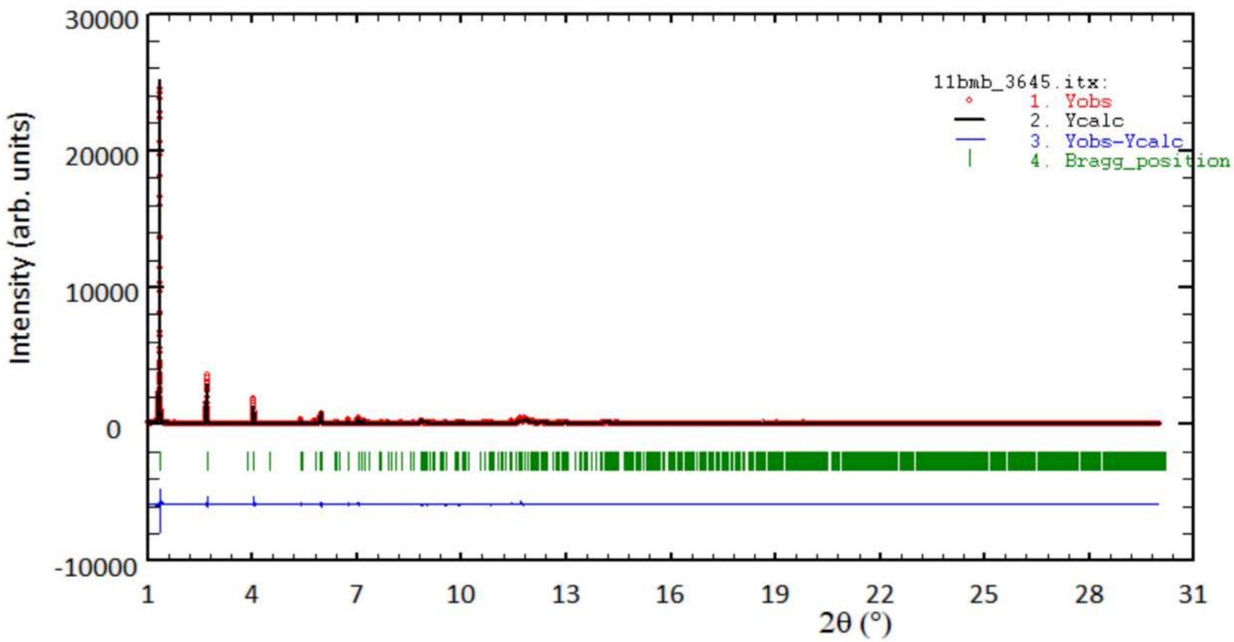

Figure S3. Rietveld with energies analysis of the structural model of **SCH_3** using XRD data. Refined structural parameters are summarized in Table S2.

Table S2. Crystal data and structural refinement for **SCH_3**. Note that refinements from Materials Studio do not provide errors for unit cell parameters.

	**SCH_3**
Empirical formula	C_7H_7SAgSe
Crystal system	Monoclinic
Space group	$P2_1/c$
a (Å)	20.2359
b (Å)	7.3026
c (Å)	5.7799
α (°)	90
β (°)	105.7226
γ (°)	90
V (Å^3)	822.2
R_p, R_{wp}	0.1549, 0.2138

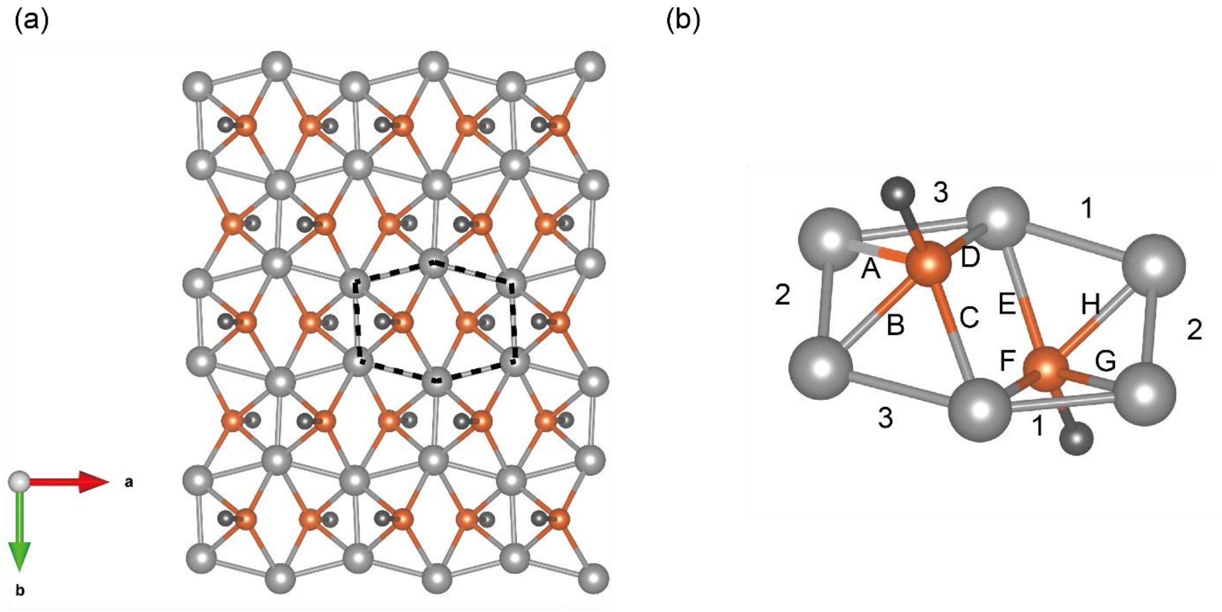

Figure S4. (a) Structure of the Ag-Se layer of AgSePh viewed from the top, showing the hexagonal pattern in a black dashed line. (b) Hexagonal pattern of AgSePh, Ag-Ag and Ag-Se bonds are labeled by 1-3 and A-H, respectively, with their values given in Table S3.

Table S3. Ag-Ag and Ag-Se bond lengths obtained from SCXRD. Bond labels are shown in Fig. S3.

Bond length (Å)	***m*F**	**H**	**CH_3**	**SCH_3**
1 (Ag-Ag)	3.1057	2.9866	2.9868	2.9990
2 (Ag-Ag)	2.9434	3.0386	2.9868	2.9990
3 (Ag-Ag)	2.8821	2.8995	2.8681	2.8569
Average (Ag-Ag)	2.913	2.975	2.947	2.952
A (Ag-Se)	2.6772	2.7012	2.6909	2.6547
B (Ag-Se)	2.6808	2.6993	2.7009	2.7391
C (Ag-Se)	2.7660	2.8155	2.6961	2.8346
D (Ag-Se)	2.8821	2.6984	2.8797	2.7101
E (Ag-Se)	2.6662	2.8596	2.6961	2.8346
F (Ag-Se)	2.7906	2.7352	2.8797	2.7101
G (Ag-Se)	2.7092	2.6885	2.6909	2.6547
H (Ag-Se)	2.7023	2.6878	2.7009	2.7391
Average (Ag-Se)	2.734	2.736	2.742	2.735

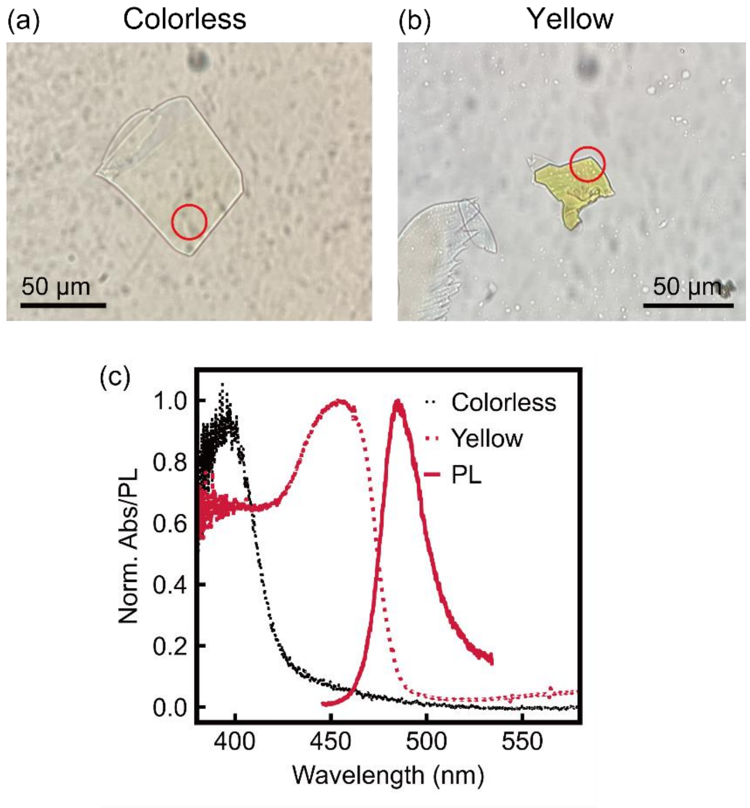

Figure S5. Optical microscope images of (a) colorless and (b) yellow crystals of **$N(CH_3)_2$**. (c) Absorption spectra of clear crystal (black, dotted) and yellow crystal (red, dotted), and PL spectrum of yellow crystal of **$N(CH_3)_2$**.

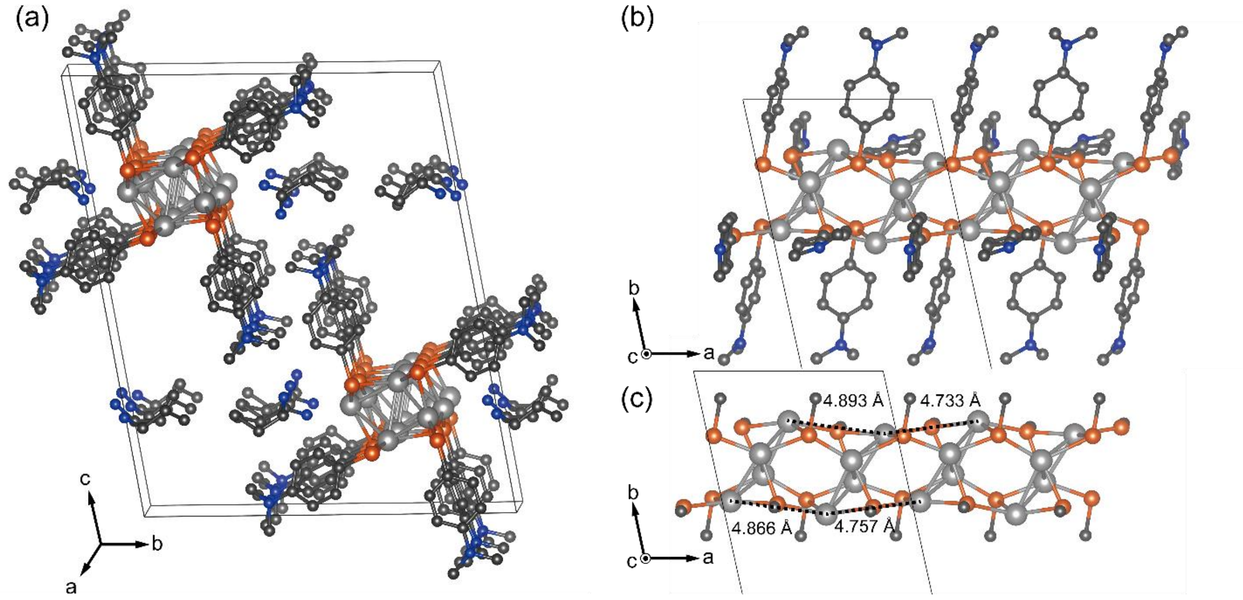

Figure S6. (a) Crystal structure of [AgSePh-$N(CH_3)_2$]$_2$·$PrNH_2$. (b) Side view of 1D chain [AgSePh-$N(CH_3)_2$]$_2$·$PrNH_2$. (c) Ag-Se core structure of [AgSePh-$N(CH_3)_2$]$_2$·$PrNH_2$.

2.3 Large-scale synthesis of MOC microcrystals

Large-scale synthesis of MOC microcrystals was developed to obtain enough materials for elemental analysis, PXRD measurement and TGA measurement. The syntheses can be scaled up to produce ~100 mg of crystals per batch while maintaining good reaction yields of 70–97.5%, with some exception of **CF_3** and ***m*F** whose yields were 40.2% and 45.4%, respectively (**Table S4**).

*Synthesis of **CF_3** and **mF***

A mixed solution of diselenide (50 mM) in toluene and $AgNO_3$ (50 mM) in 1-butylamine was filtered through a PTFE syringe filter. Then, 10 mL of the filtered solution was transferred to a 20 mL glass vial, which was placed on deionized water to allow water diffusion at room temperature over 6 days. The yields were 33 mg of **CF_3** (40.2%) and 31 mg of ***m*F** (45.4%).

*Synthesis of **SCH_3***

Two solutions, each 20 mL, containing diselenide (40 mM) and $AgNO_3$ (40 mM) in 1-butylamine, were prepared and cooled to 5 °C before being mixed. The combined solution was then maintained at 5 °C overnight. The resulting precipitate was collected by suction filtration, washed with 2-propanol and toluene, and dried under vacuum overnight to yield 0.222 g (89.5%).

*Synthesis of **CH_3**, **H**, **pF**, **OC_8H_{17}**, and **Napht**,*

A 20 mL solution of diselenide (40 mM) in toluene was mixed with a 20 mL solution of $AgNO_3$ (40 mM) in 1-butylamine at room temperature. The combined solution was then kept at room temperature overnight. The resulting precipitate was collected by suction filtration, washed with 2-propanol and toluene, and dried under vacuum overnight. The yields of the obtained solids were as follows: **CH_3** (0.196 g, 88.1%), **H** (0.178 g, 84.3%), **pF** (0.160 g, 70.9%), **OC_8H_{17}** (0.270 g, 86.1%), and **Napht** (0.245 g, 97.5%).

Table S4. Results of large-scale synthesis and elemental analysis of MOCs.

	Yield	Elemental analysis	
		Calcd.	Found
**CF_3**	40.2%	C 25.33%, H 1.21%	C 26.48%, H 1.51%
***m*F**	45.4%	C 25.56%, H 1.43%	C 25.87%, H 1.26%
**H**	84.3%	C 30.24%, H 2.54%	C 27.37%, H 1.85%
**CH_3**	88.1%	C 27.30%, H 1.91%	C 30.44%, H 2.31%
***p*F**	70.9%	C 25.56%, H 1.43%	C 25.67%, H 1.16%
**OC_8H_{17}**	86.1%	C 42.87%, H 5.40%	C 42.68%, H 5.39%
**SCH_3**	89.5%	C 27.11%, H 2.28%	C 27.35%, H 1.96%
**Napht**	97.5%	C 38.25%, H 2.25%	C 39.25%, H 2.03%

3. Supplemental Figures and Tables

Table S5. Peak positions of PXRD patterns and calculated periodicity of interlayer distance.

	2θ [°]			Periodicity of 2θ [°][a]	Periodicity [nm]
	Peak 1	Peak 2	Peak 3		
**CF_3**	4.944	9.832	14.733	4.89	1.81
***m*F**	5.847	11.687	17.551	5.85	1.51
**H**	6.140	12.237	18.373	6.12	1.44
**CH_3**	5.113	10.235	15.383	5.14	1.72
***p*F**	5.847	11.647	17.471	5.81	1.52
**OC_8H_{17}**	5.143	7.735	10.335	2.60	3.40
**SCH_3**	4.476	9.013	13.563	4.54	1.94
**Napht**	8.991	13.599	18.223	4.62	1.91

a: periodicity of 2θ was the average of (peak 2 – peak 1) and (peak 3 – peak 2).

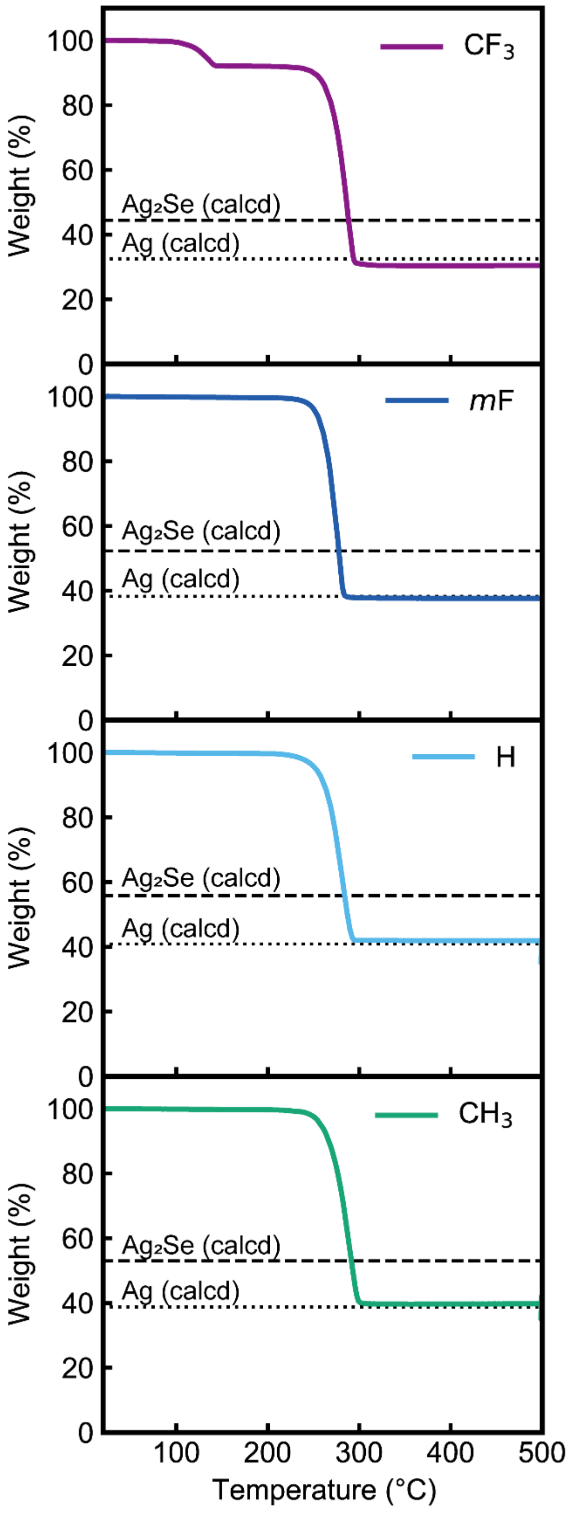

Figure S7. TGA curves of MOCs under N_2 flow. Calculated contents of Ag and Ag_2Se are shown as dotted and dashed lines, respectively.

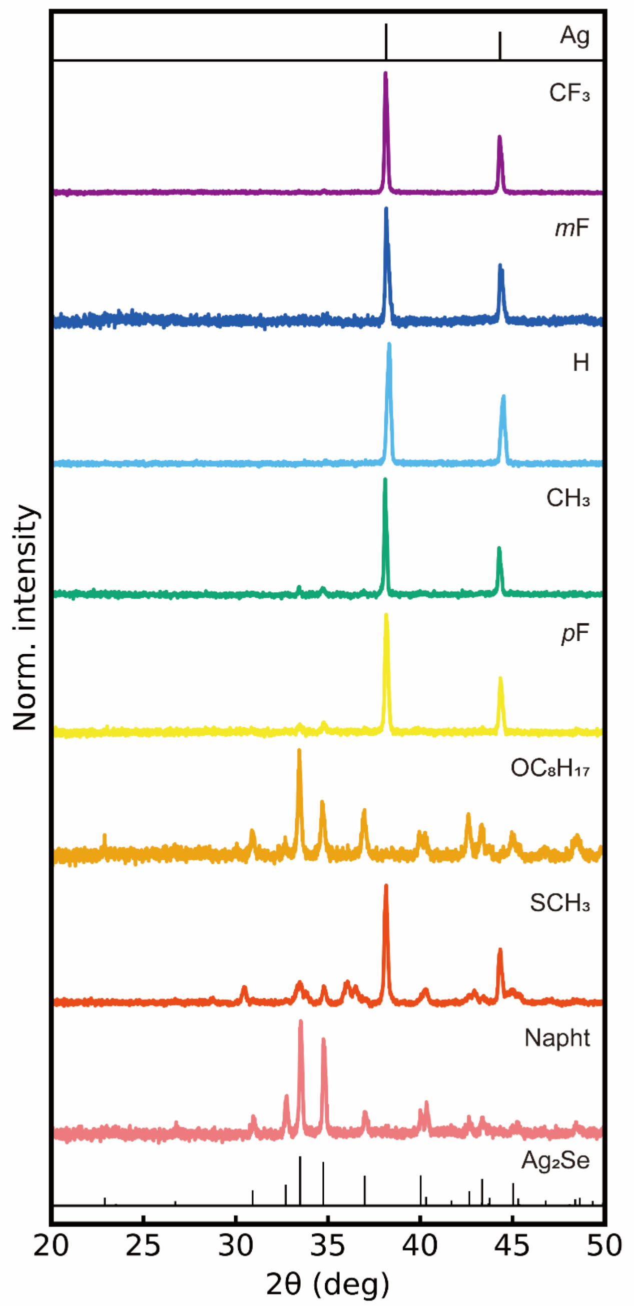

Figure S8. PXRD patterns of residues after TGA measurements at 500 °C under N_2 flow. PXRD patterns of Ag and Ag_2Se (orthorhombic, ICSD No. 15213) were shown as references.

Table S6. Decomposition temperatures from TGA measurements and the residue weights at 500°C.

R=	Decomposition temperature at X% weight loss [°C]			Weight at 500 °C [wt%]	Calculated Content [wt%]	
	X = 1%	5%	10%		Ag	Ag_2Se
**CF_3**	108	132	250	30.5	**32.5**	44.4
***m*F**	233	253	259	37.7	**38.3**	52.3
**H**	227	252	262	41.9	**40.9**	55.8
**CH_3**	238	258	267	39.7	**38.8**	53.0
***p*F**	234	255	264	40.0	**38.3**	52.3
**OC_8H_{17}**	229	249	260	37.1	27.5	**37.6**
**SCH_3**	217	266	275	41.7	34.8	47.5
**Napht**	258	294	302	46.2	34.4	**46.9**

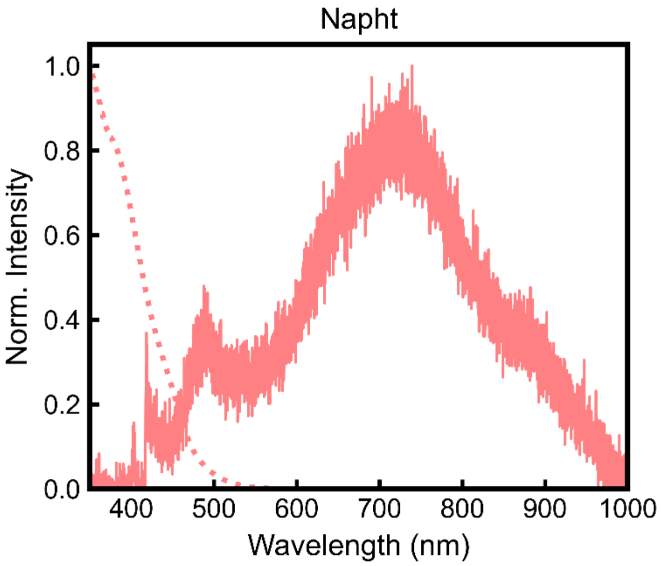

Figure S9. Diffuse reflectance UV-Vis (dashed) and PL (solid) spectra **Napht** crystals.

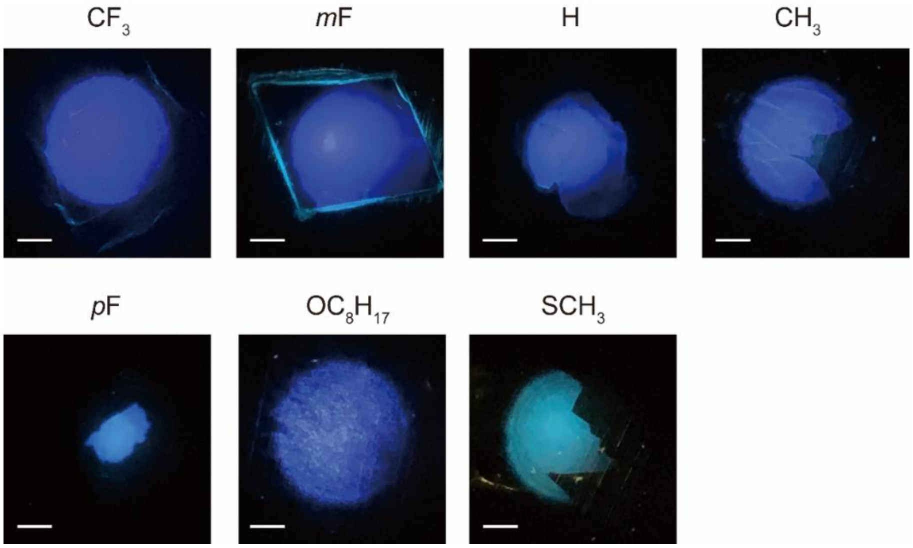

Figure S10 Optical micrographs of MOC crystals under UV light (405 nm). Scale bars = 100 μm.

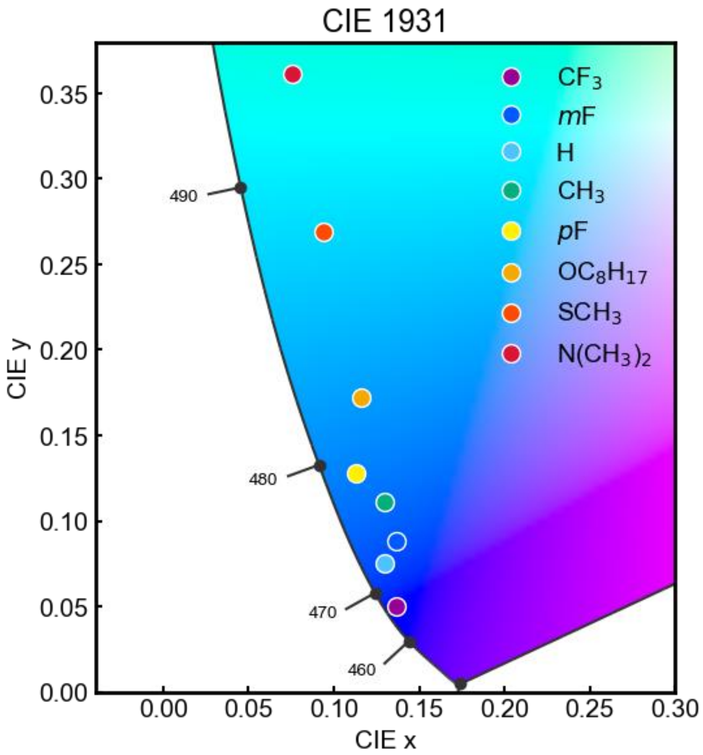

Figure S11. CIE 1931 [x, y] coordinates of synthesized MOCs.

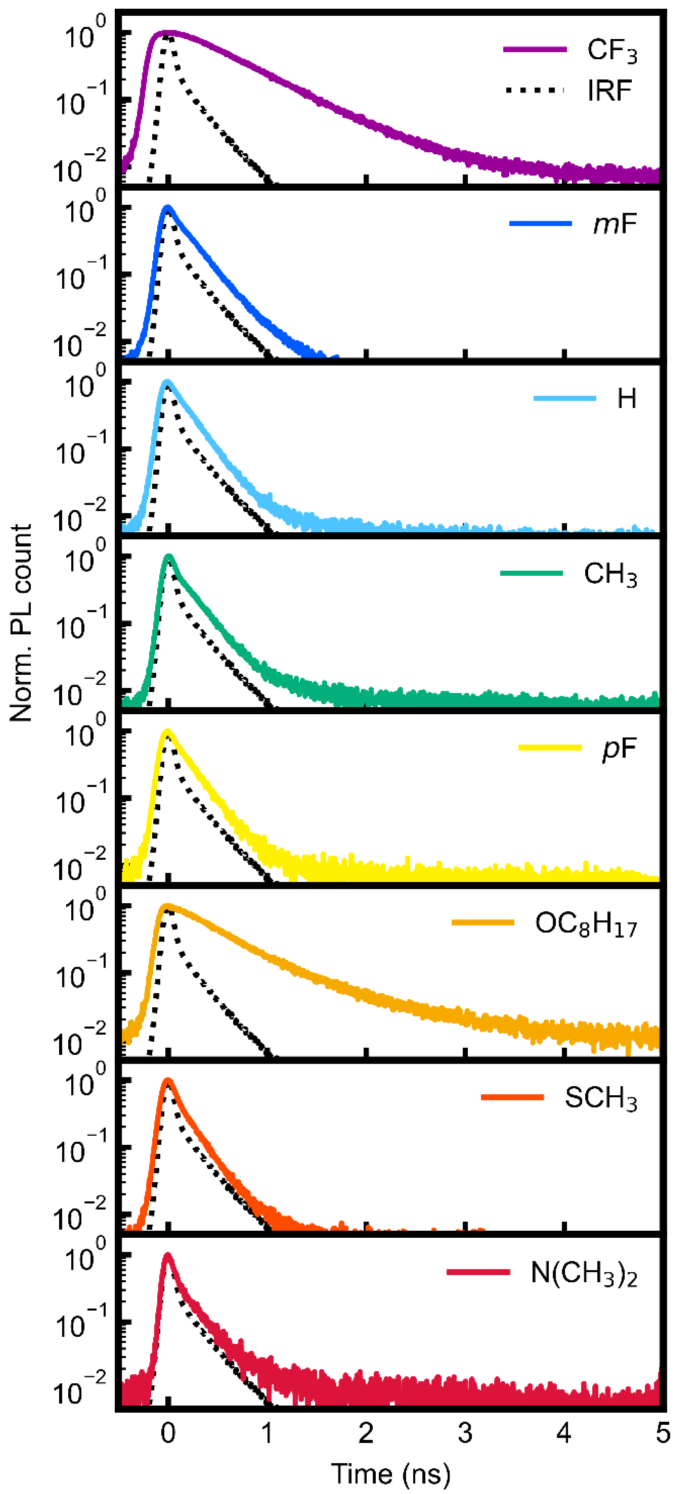

Figure S12. Time-resolved PL decays of synthesized MOCs and instrument response function (IRF).

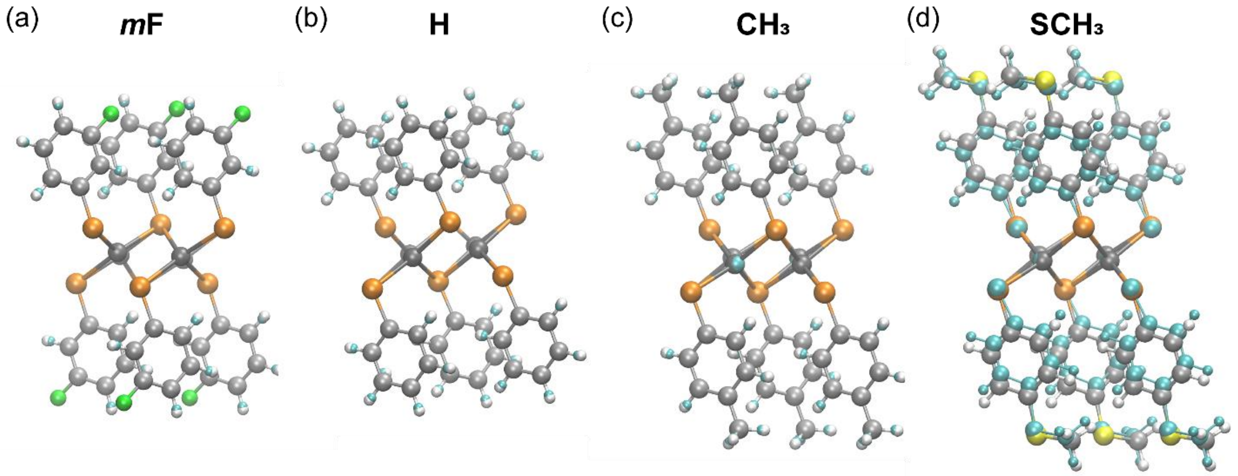

Figure S13. Overlaid experimentally obtained and DFT-optimized structures for (a) ***m*F**, (b) **H**, (c) **CH_3**, and (d) **SCH_3**. The experimentally obtained structures are shown in cyan, and the DFT-optimized structures are shown with atoms colored as follows: Ag = dark gray, Se = orange, C = light gray, F = green, S = yellow, and H = white. In cases where the structures are nearly identical, only the DFT-optimized structures are visible.

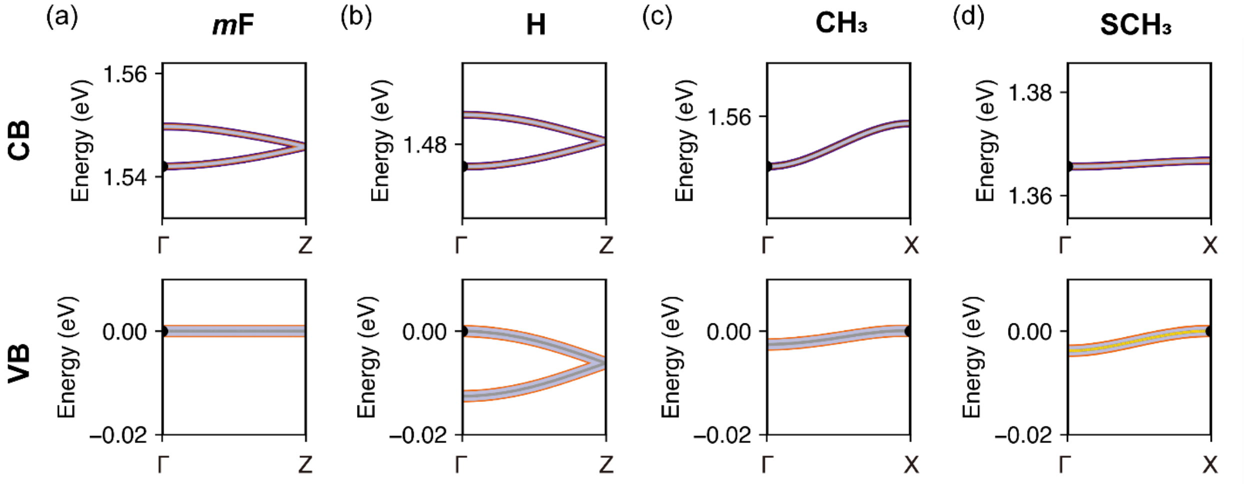

Figure S14. Electronic band structure of (a) ***m*F**, (b) **H**, (c) **CH_3**, (d) **SCH_3** zoomed to the band edge of the valence band (VB) and the conduction band (CB). The band thickness represents the contribution from each orbital, following the same color scheme as in Figure S14.

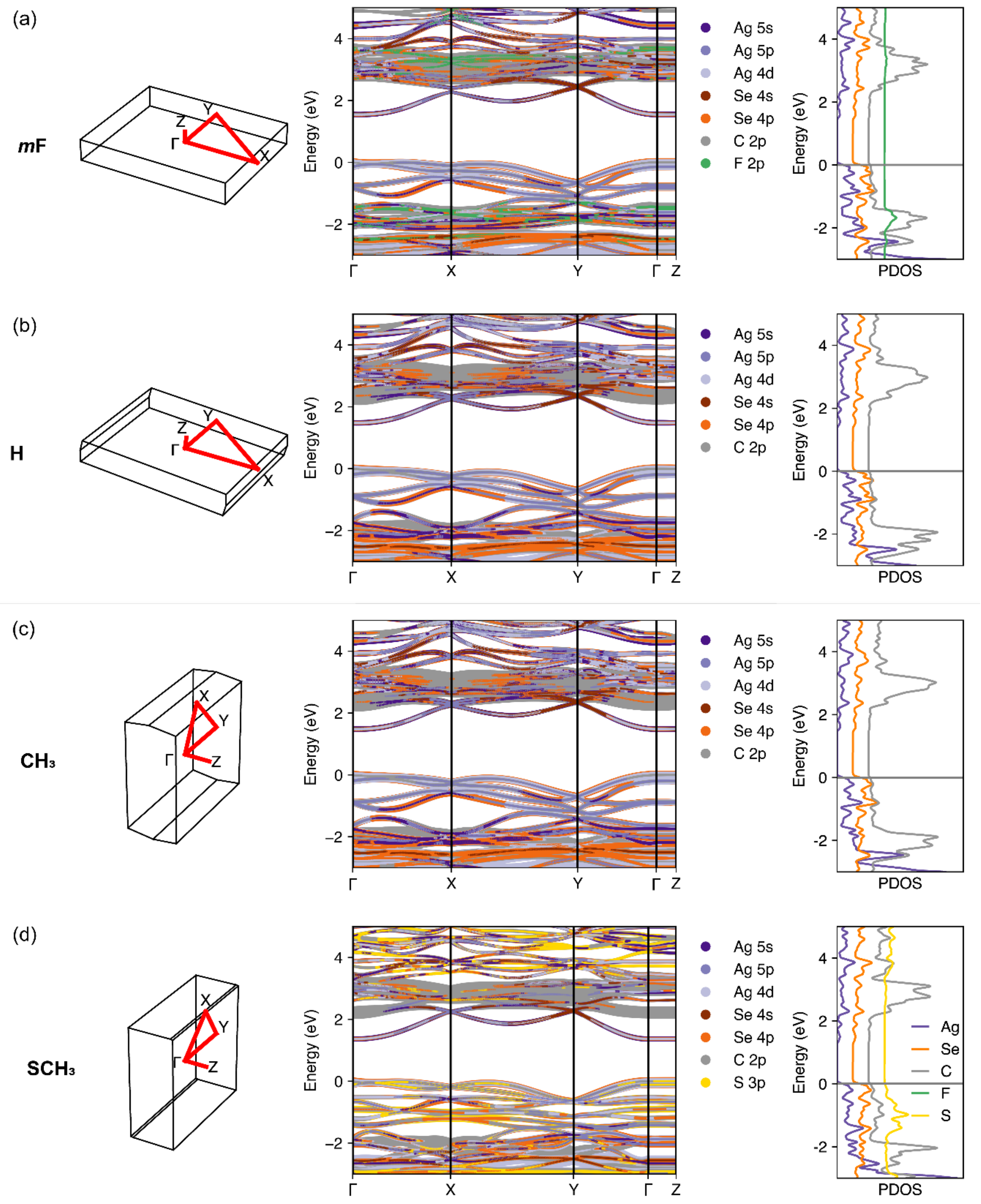

Figure S15. The first Brillouin zone (left), k-dependent projected density of states (PDOS) (center), and Brillouin zone-integrated PDOS (right) for (a) ***m*F**, (b) **H**, (c) **CH_3**, and (d) **SCH_3**, calculated using DFT with the PBE functional. The k-path chosen for the band structure plot is indicated by red lines in the first Brillouin zone. Contributions from Ag (purple), Se (orange), C (gray), F (green), and S (yellow), where applicable, are colored as also shown in inset legend. In the k-dependent PDOS, the band thickness represents the contribution from each orbital, with the orbital exhibiting the highest contribution plotted first, followed by those with lower contribution, layered in decreasing order of contribution.

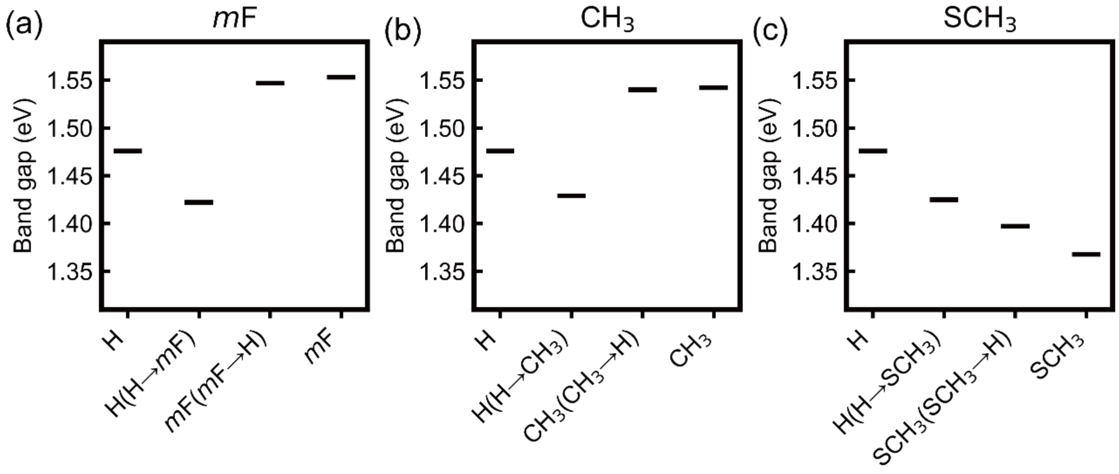

Figure S16. Direct band gap changes resulting from modifying either the functional group or the inorganic structure framework for (a) ***m*F**, (b) **CH_3**, and (c) **SCH_3**. A(A→B) denotes the band gap of a MOC with the inorganic framework of A with the functional group substituted by B. When A≠B, geometry was not re-optimized, but interlayer distances were adjusted to avoid unphysical atomic overlaps.

¹H NMR (400 MHz, $CDCl_3$) of 1,2-bis(4-trifluoromethylphenyl) diselenide

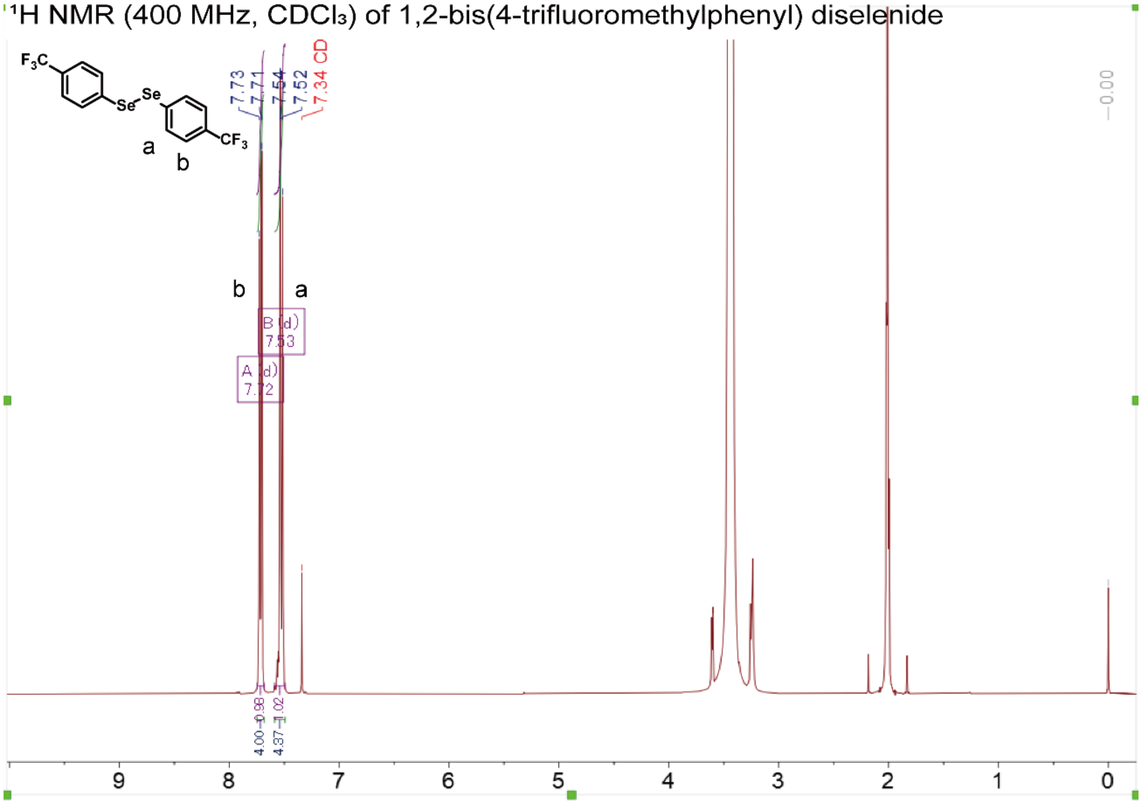

¹³C NMR (101 MHz, $CDCl_3$) of 1,2-bis(4-trifluoromethylphenyl) diselenide

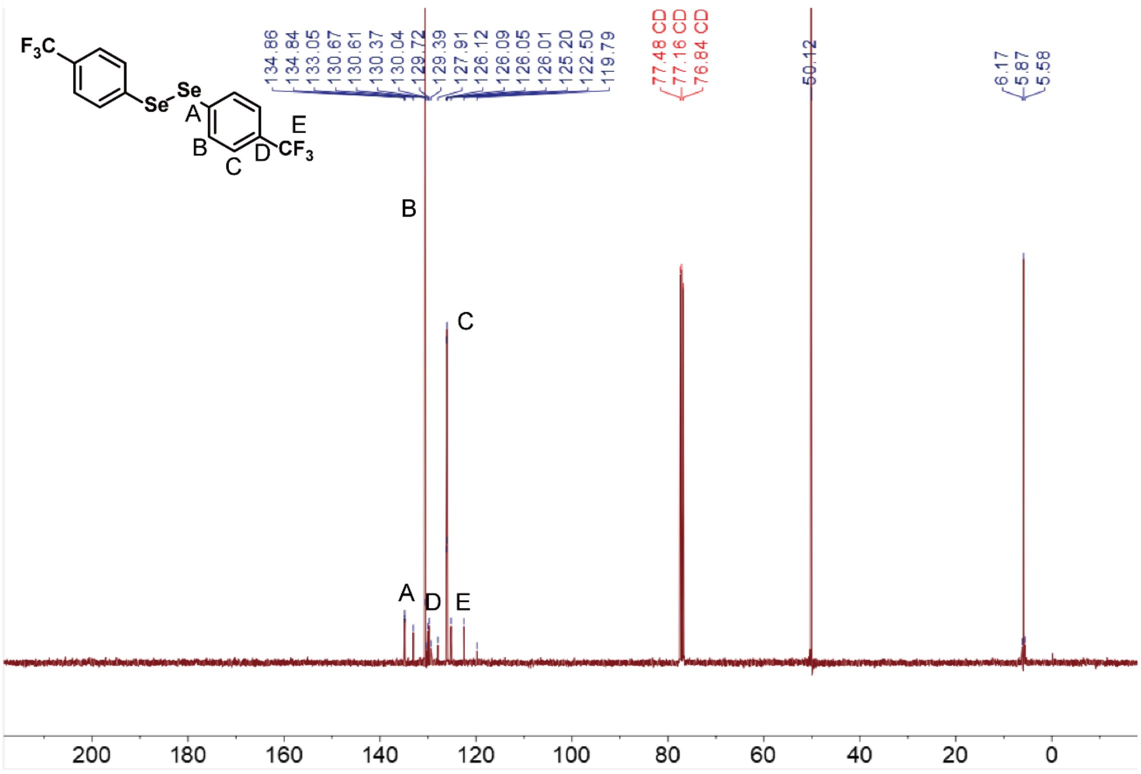

^{19}F NMR (376 MHz, $CDCl_3$) of 1,2-bis(4-trifluoromethylphenyl) diselenide

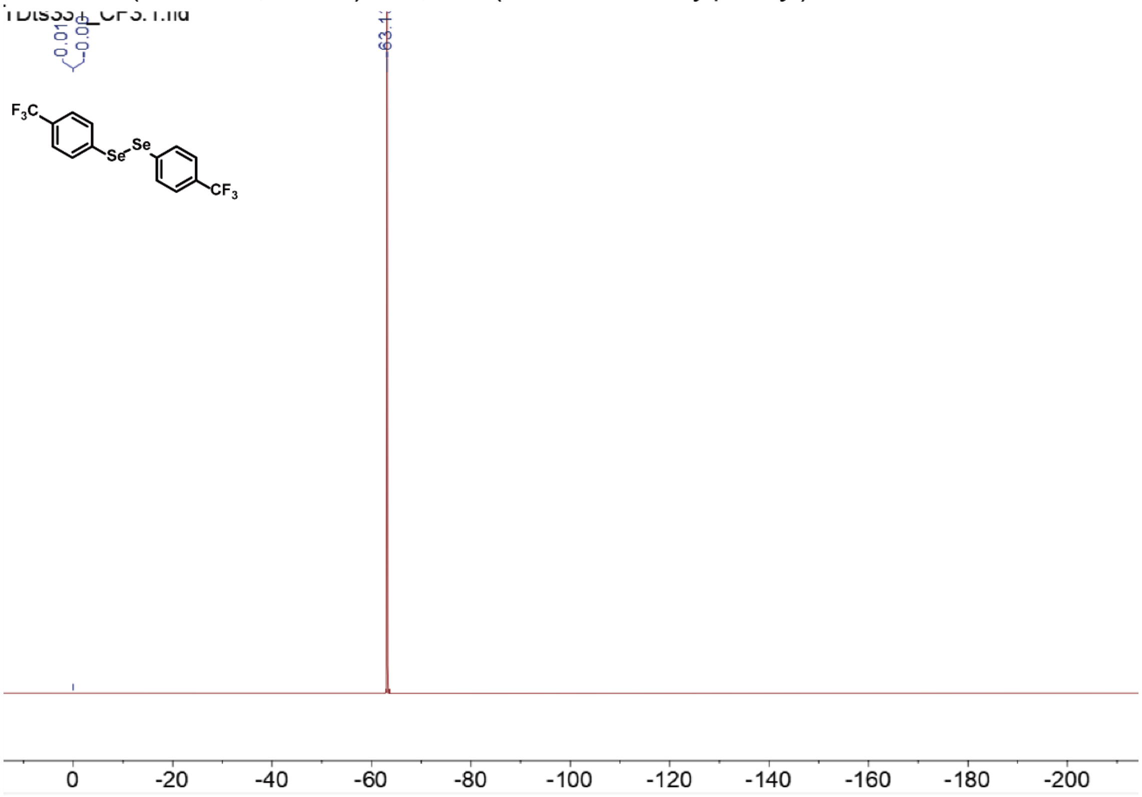

^{77}Se NMR (101 MHz, $CDCl_3$) of 1,2-bis(4-trifluoromethylphenyl) diselenide

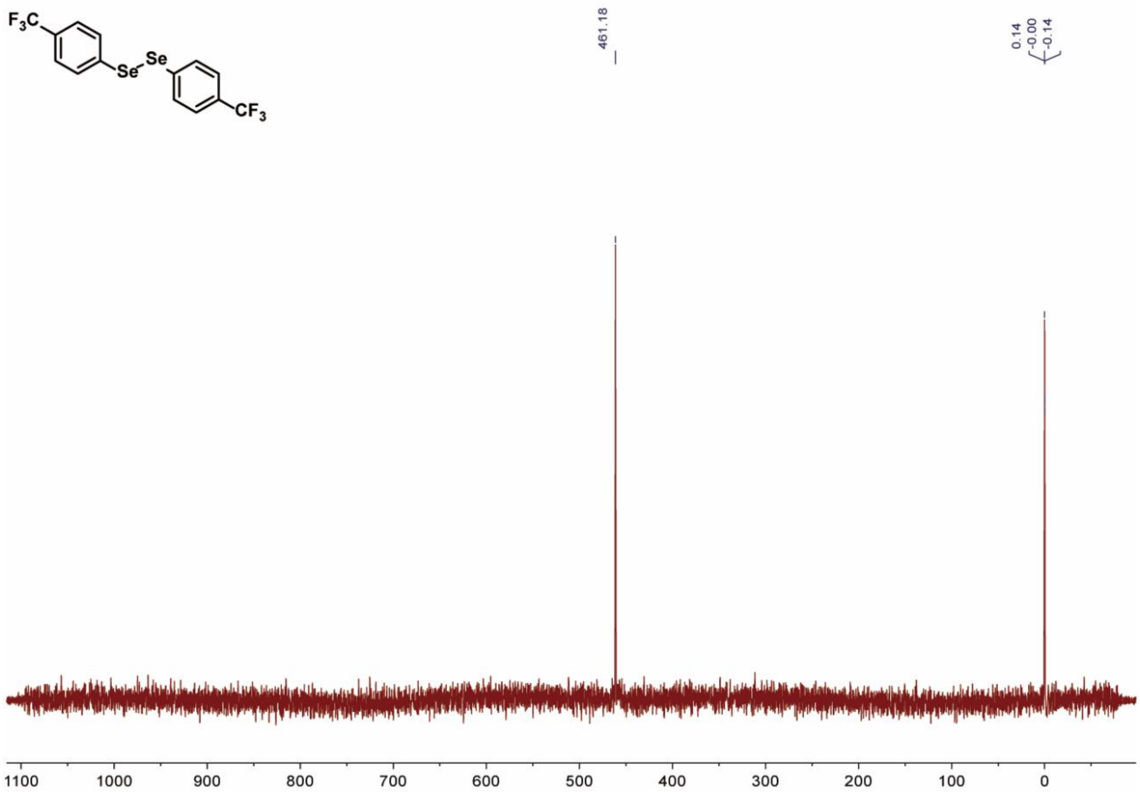

^1H NMR (400 MHz, $CDCl_3$) of 1,2-bis(3-fluorophenyl) diselenide

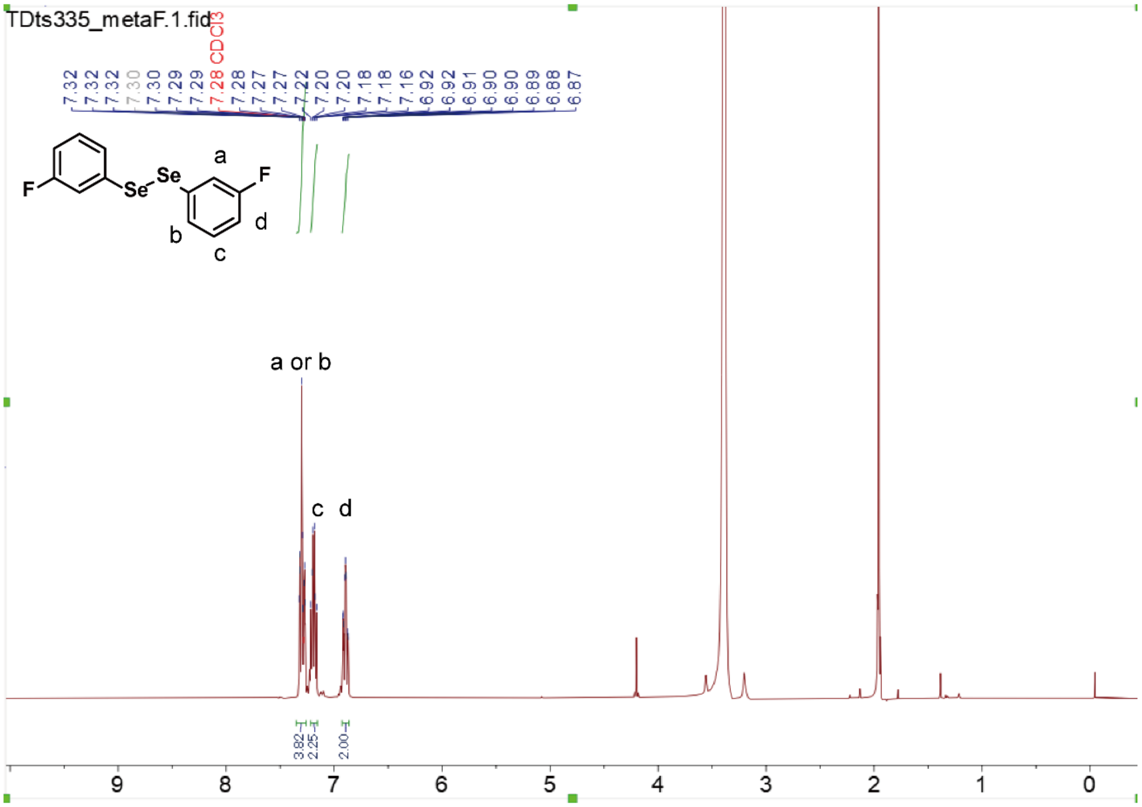

^{19}F NMR (376 MHz, $CDCl_3$) of 1,2-bis(3-fluorophenyl) diselenide

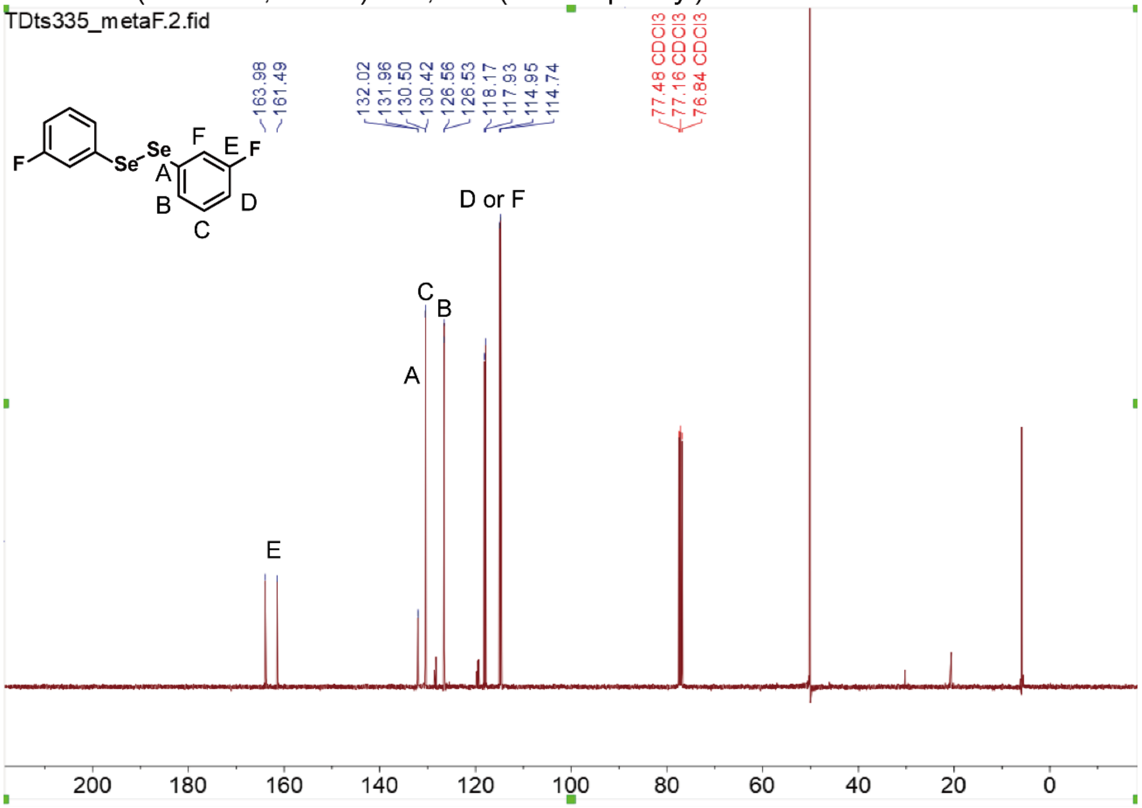

^{19}F NMR (376 MHz, $CDCl_3$) of 1,2-bis(3-fluorophenyl) diselenide

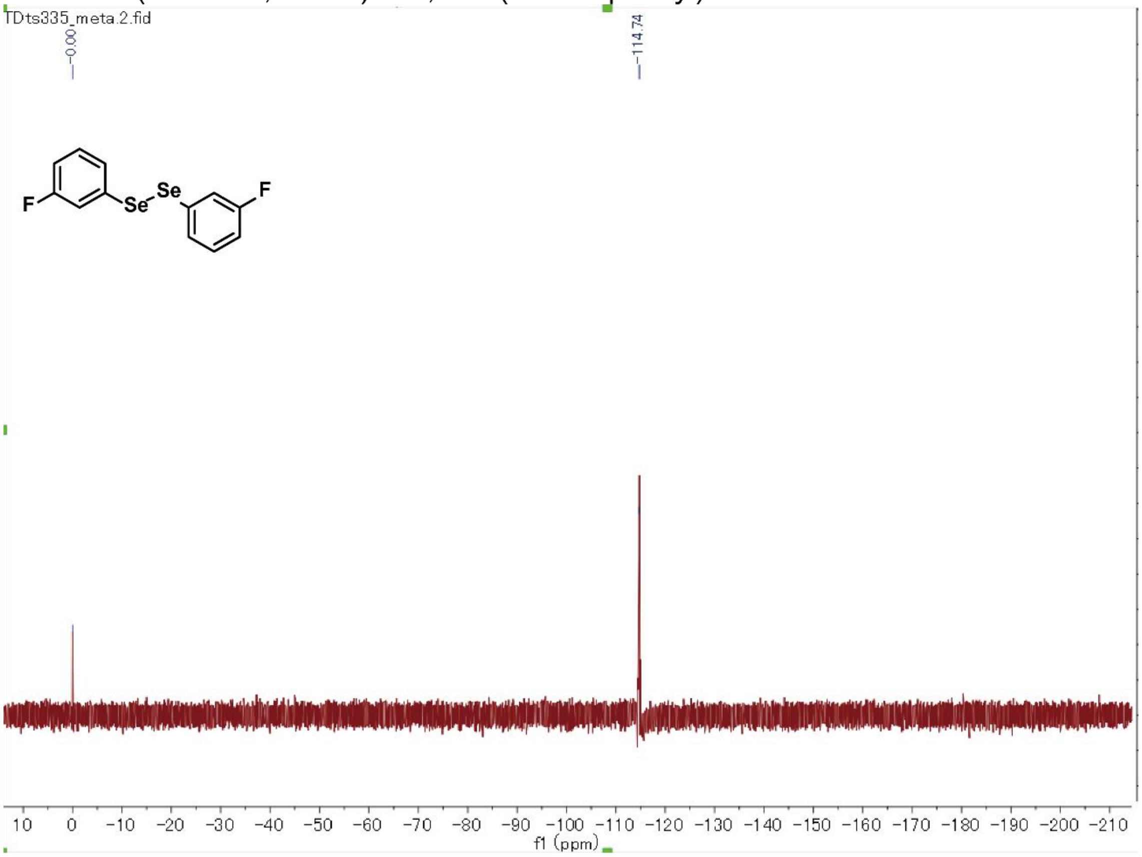

^{77}Se NMR (101 MHz, $CDCl_3$) of 1,2-bis(3-fluorophenyl) diselenide

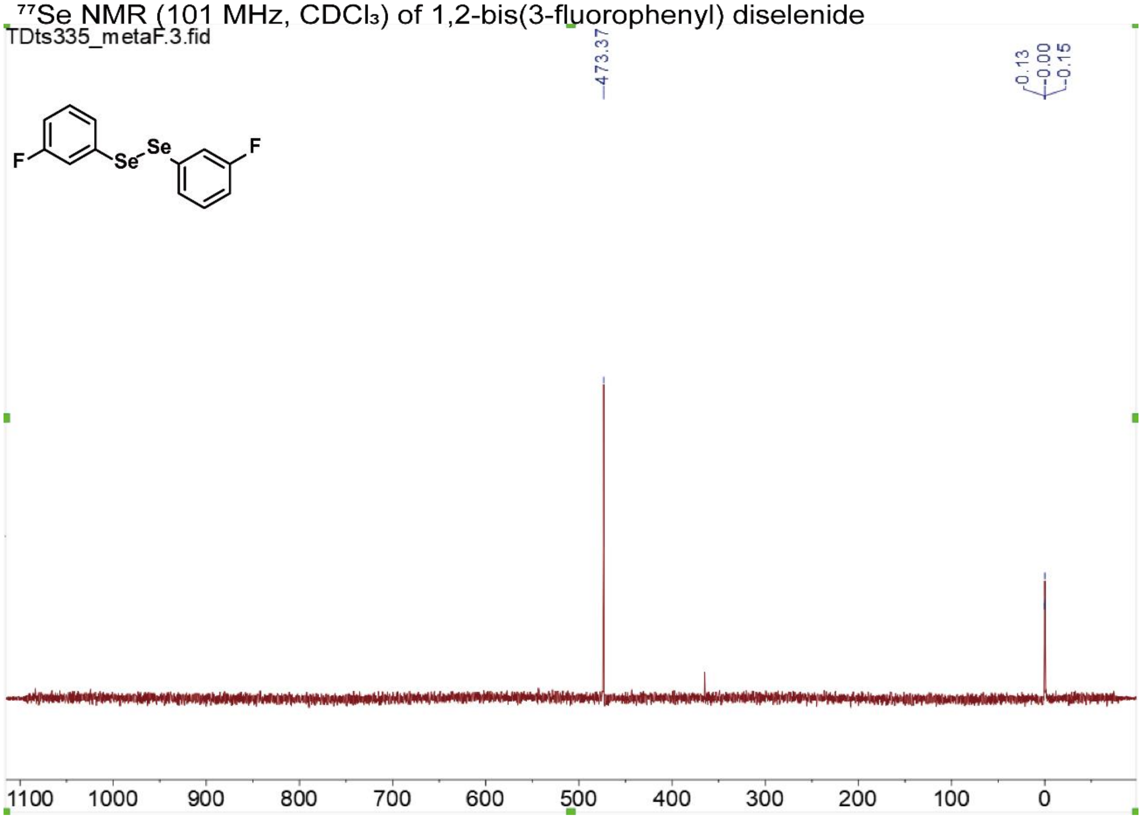

^{1}H NMR (400 MHz, $CDCl_3$) of 1,2-bis(4-methylphenyl) diselenide

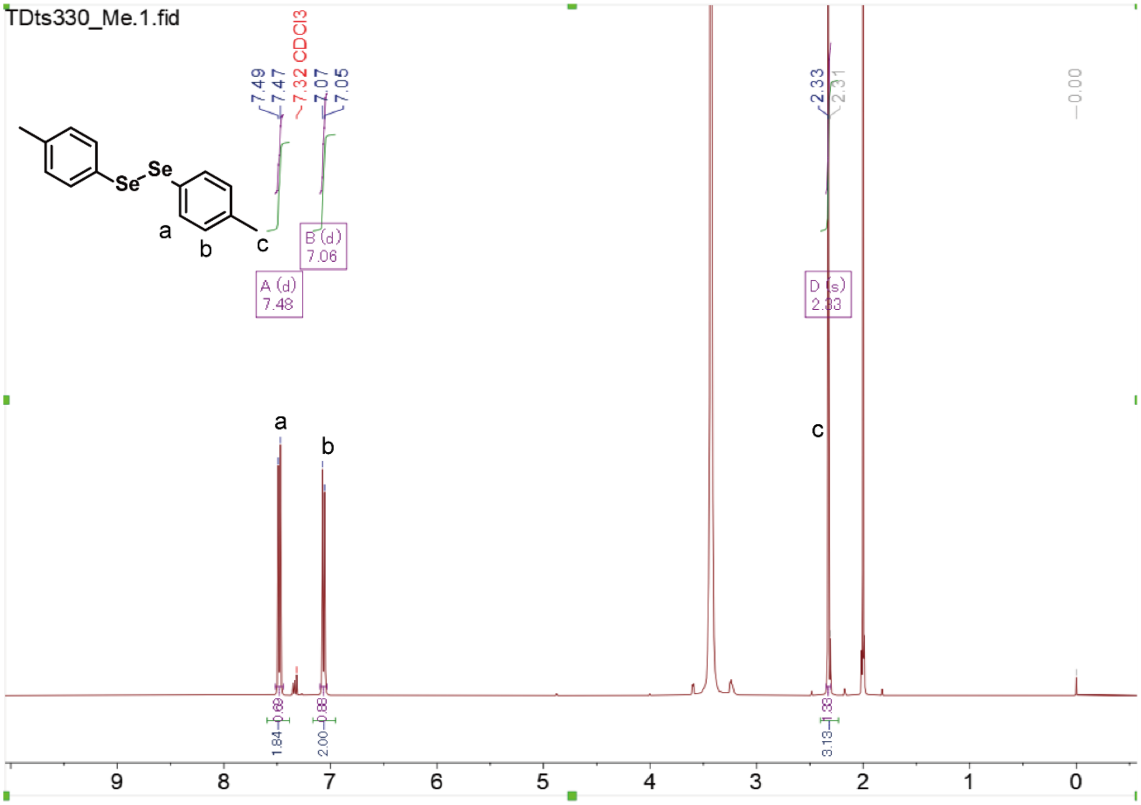

^{13}C NMR (101 MHz, $CDCl_3$) of 1,2-bis(4-methylphenyl) diselenide

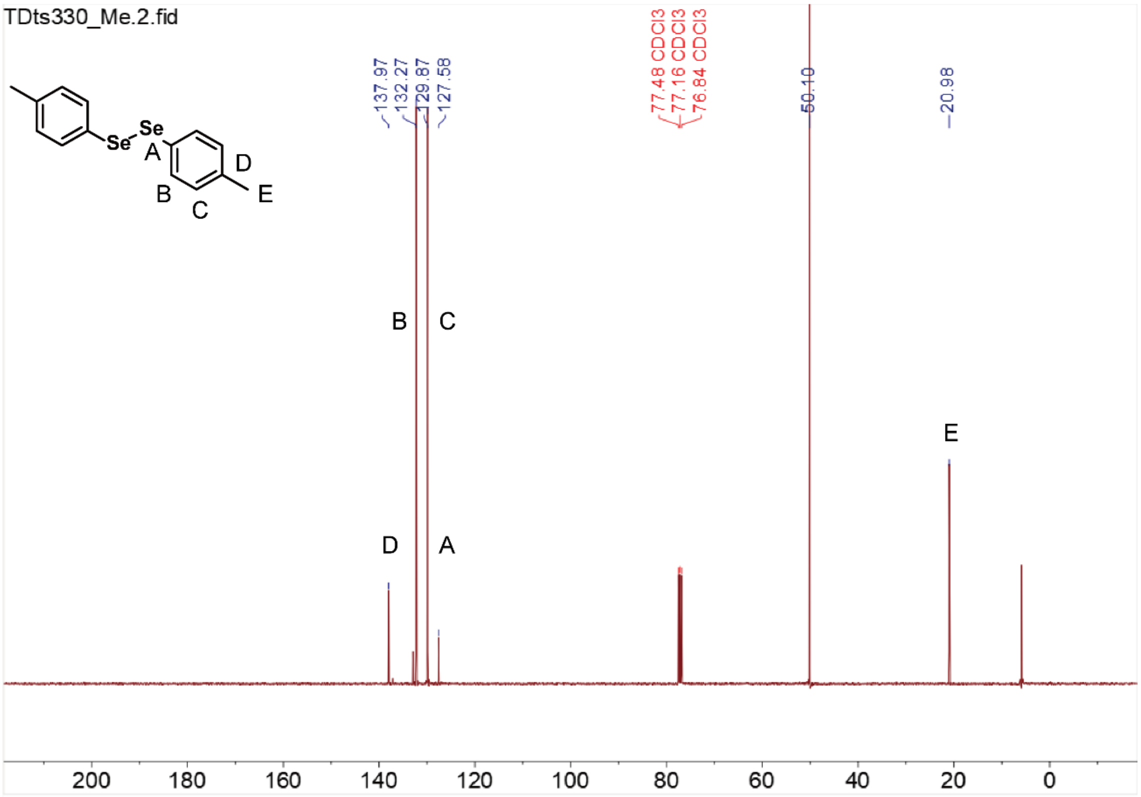

^{77}Se NMR (101 MHz, $CDCl_3$) of 1,2-bis(4-methylphenyl) diselenide

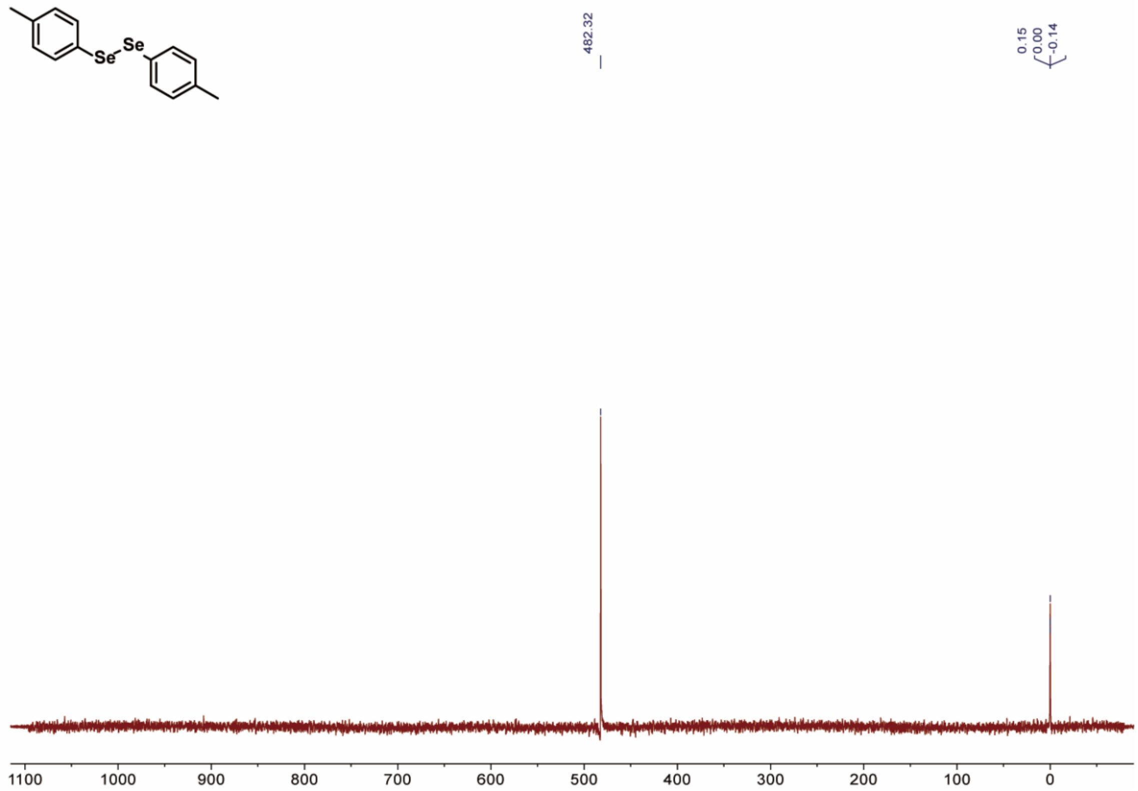

¹H NMR (400 MHz, $CDCl_3$) of 1,2-bis(4-fluorophenyl) diselenide

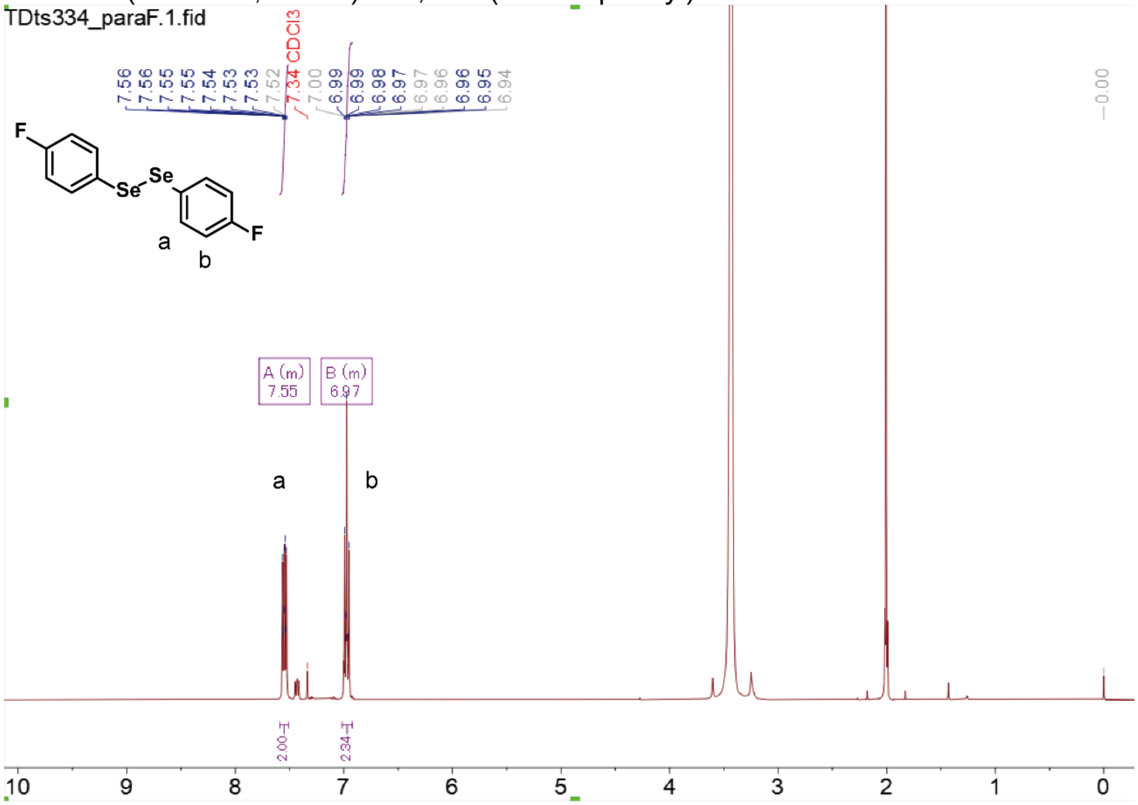

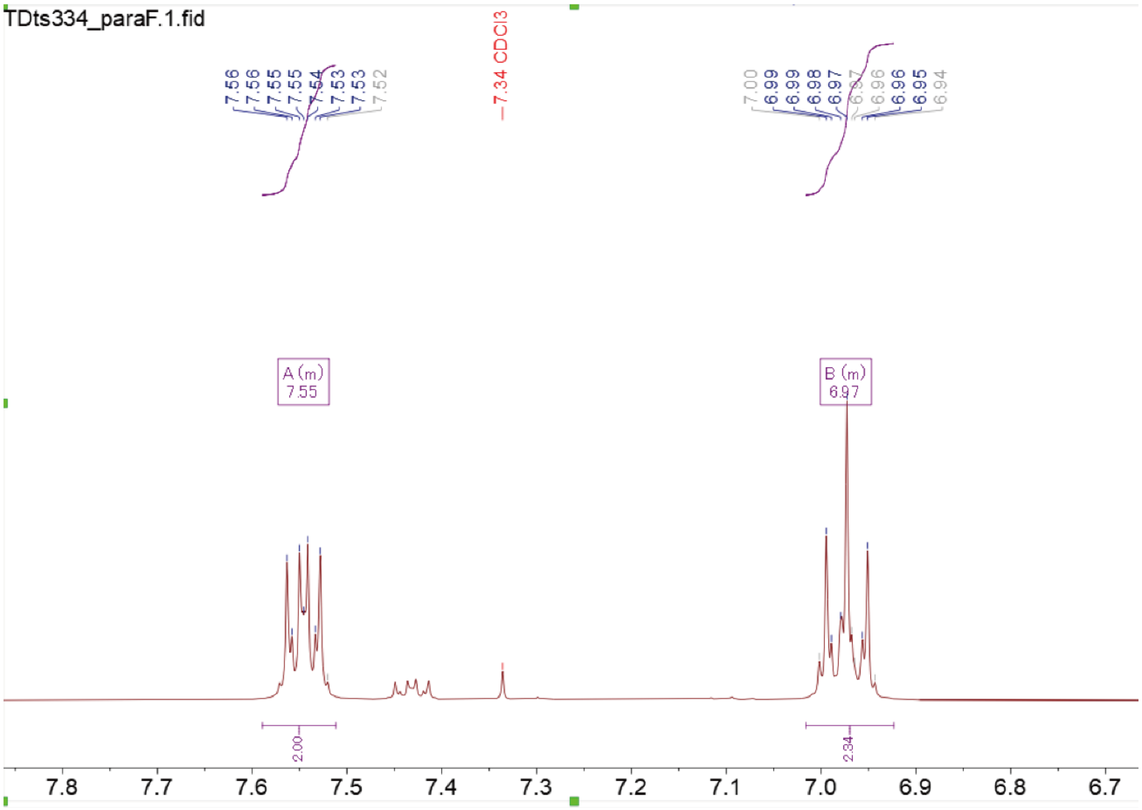

¹³C NMR (101 MHz, $CDCl_3$) of 1,2-bis(4-fluorophenyl) diselenide

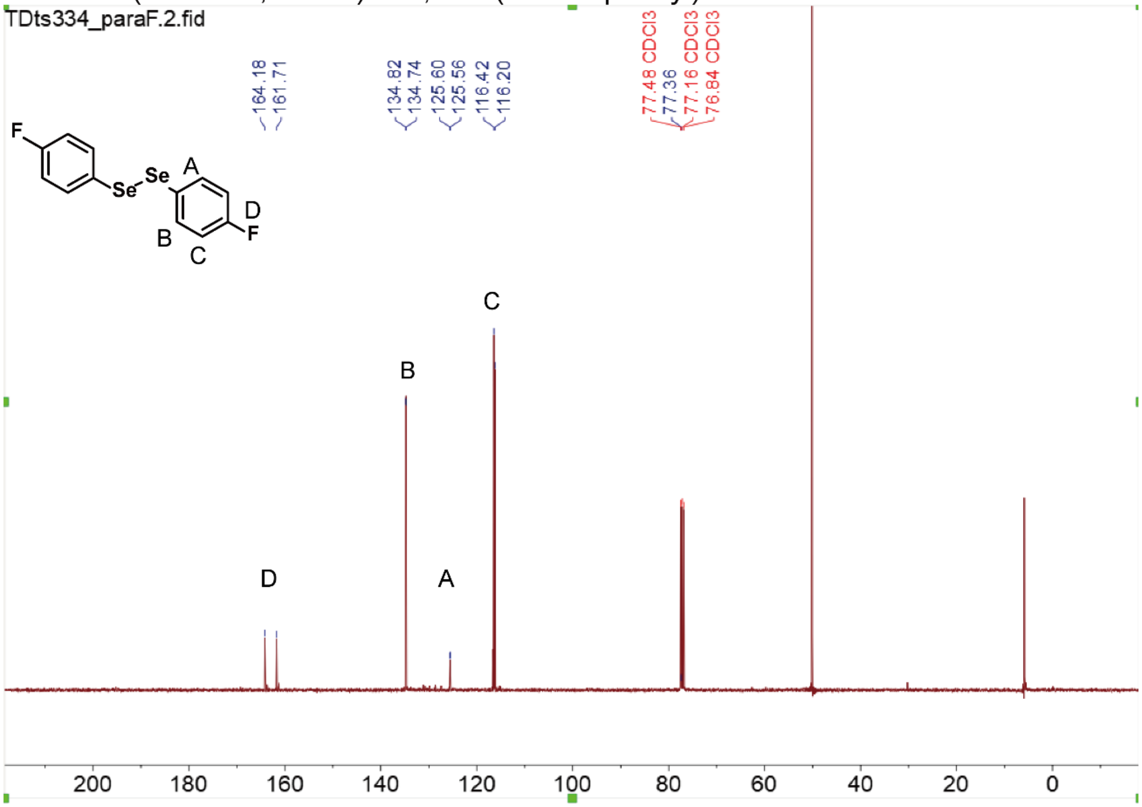

¹⁹F NMR (376 MHz, $CDCl_3$) of 1,2-bis(4-fluorophenyl) diselenide

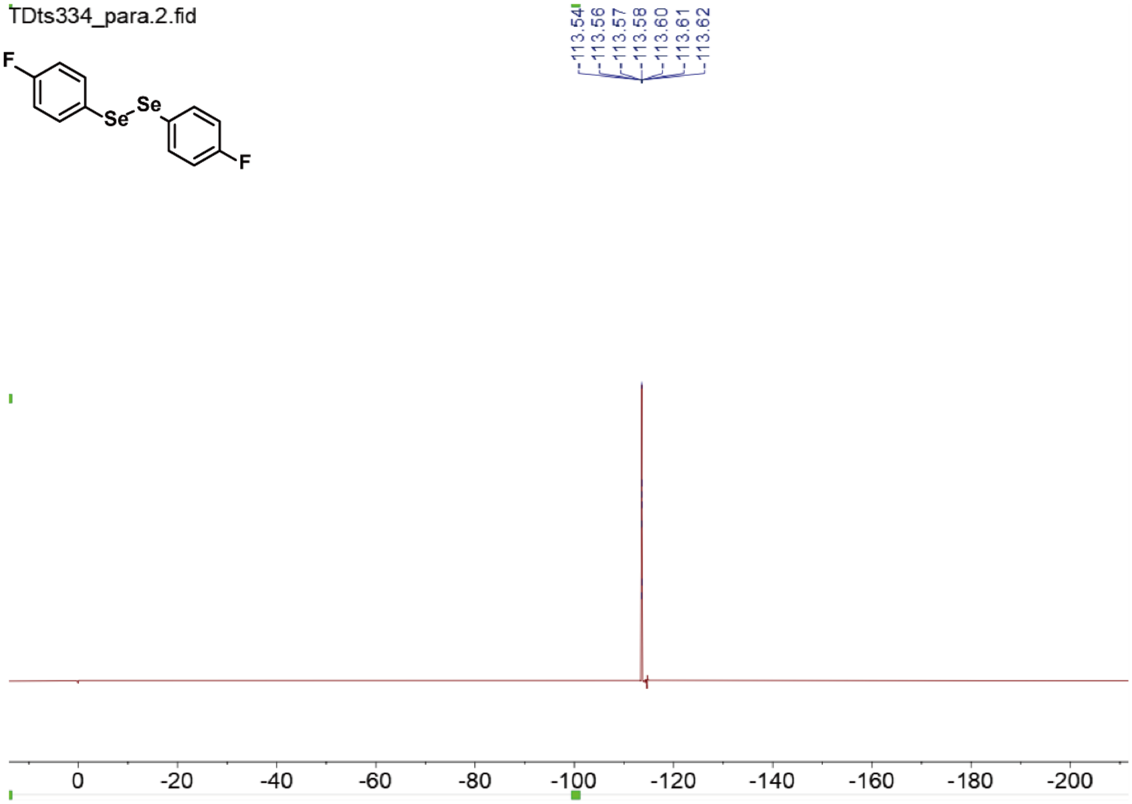

^{77}Se NMR (101 MHz, $CDCl_3$) of 1,2-bis(4-fluorophenyl) diselenide

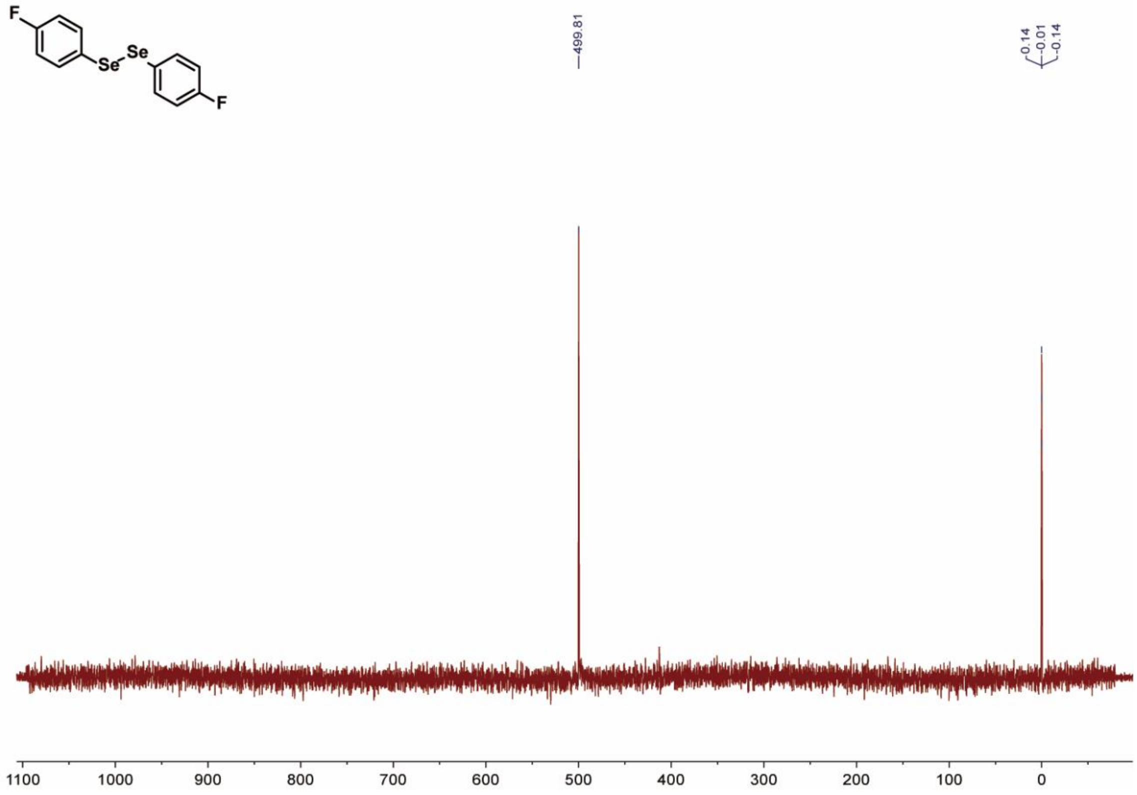

^1H NMR (400 MHz, $CDCl_3$) of 1,2-bis(4-octoxyphenyl) diselenide

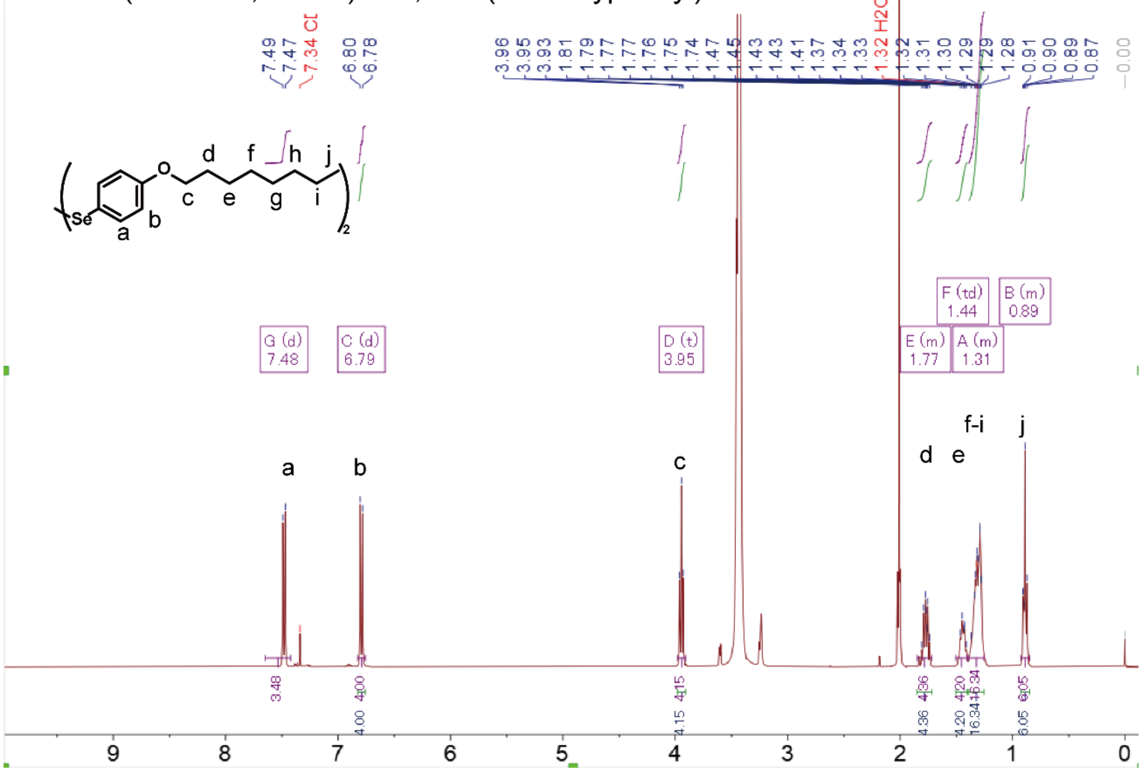

^{13}C NMR (101 MHz, $CDCl_3$) of 1,2-bis(4-octoxyphenyl) diselenide

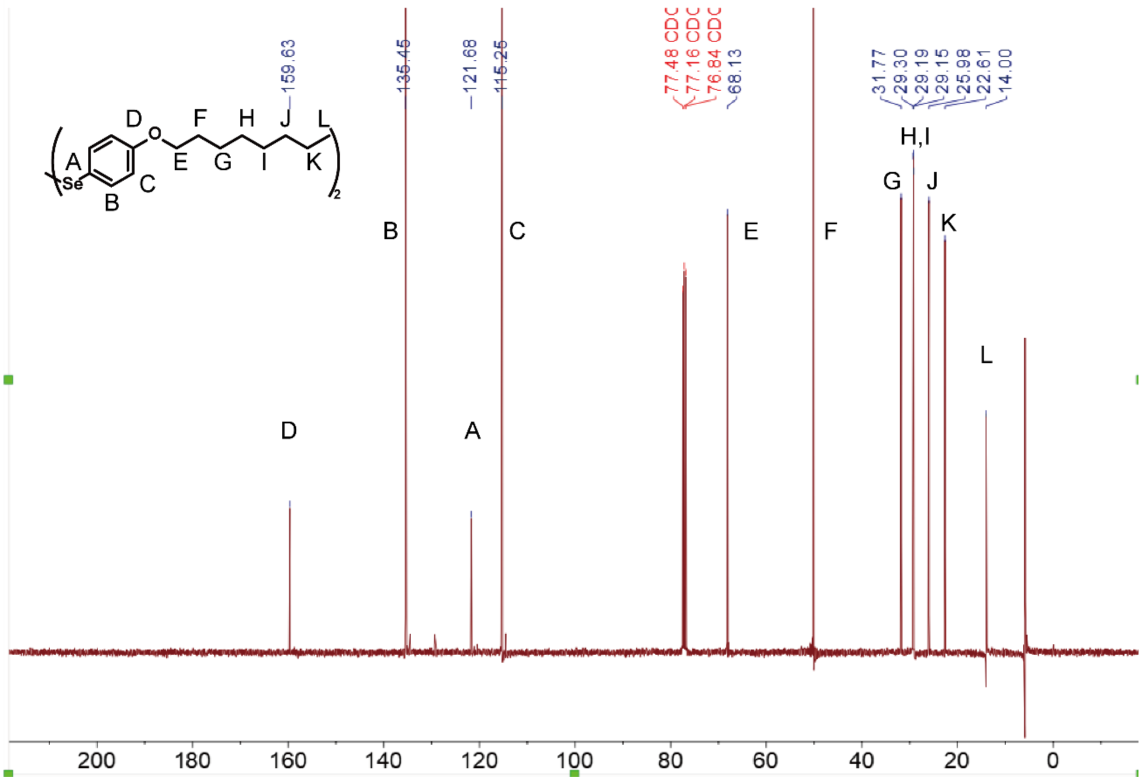

77Se NMR (101 MHz, CDCl₃) of 1,2-bis(4-octoxyphenyl) diselenide

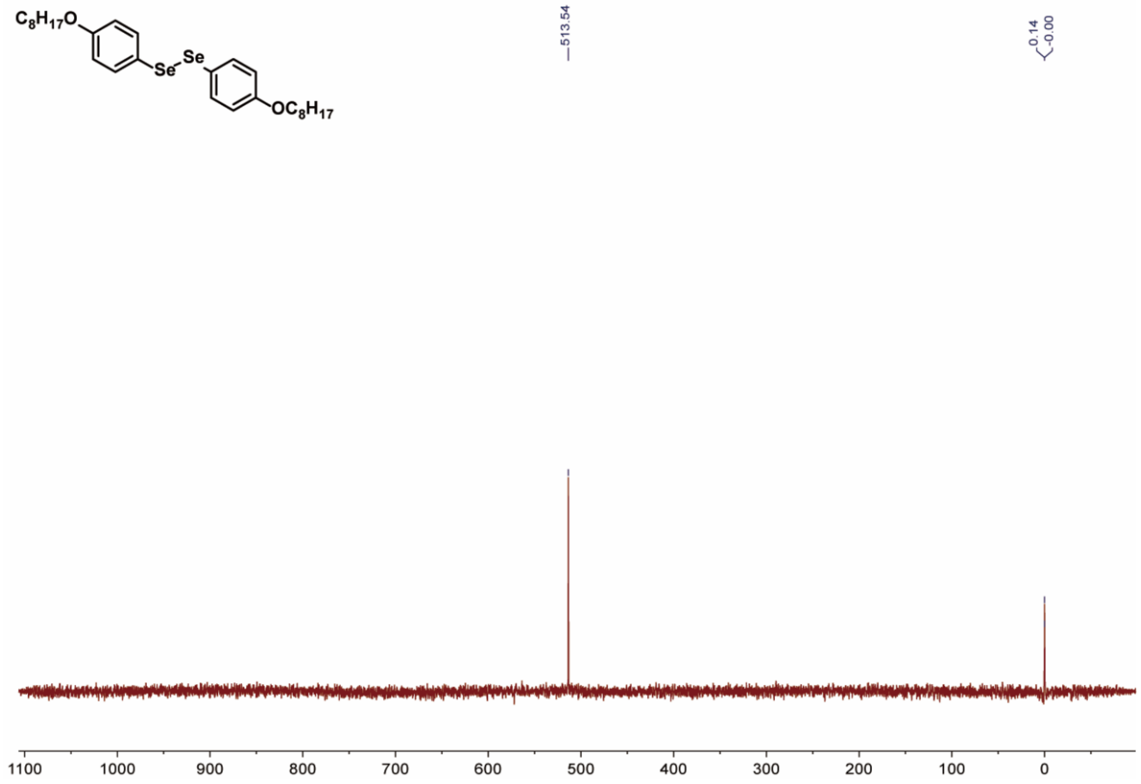

^{1}H NMR (400 MHZ, $CDCl_3$) of 1,2-bis(4-thiomethylphenyl) diselenide

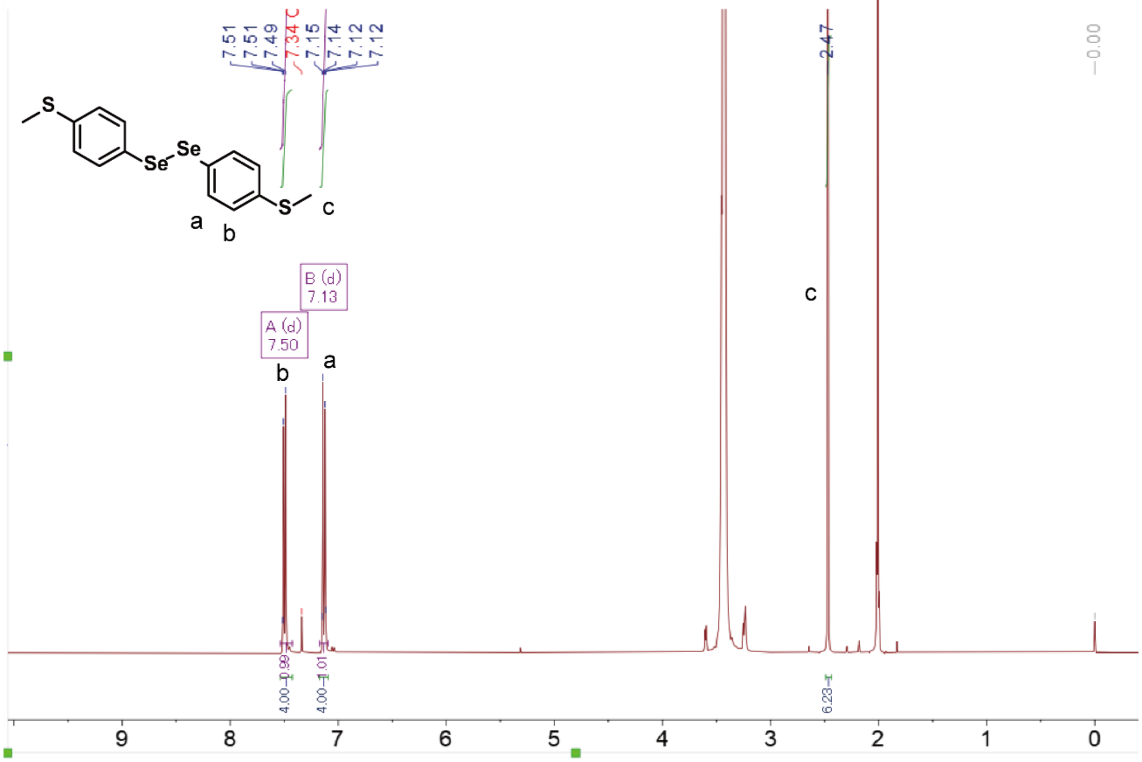

^{13}C NMR (101 MHz, $CDCl_3$) of 1,2-bis(4-thiomethylphenyl) diselenide

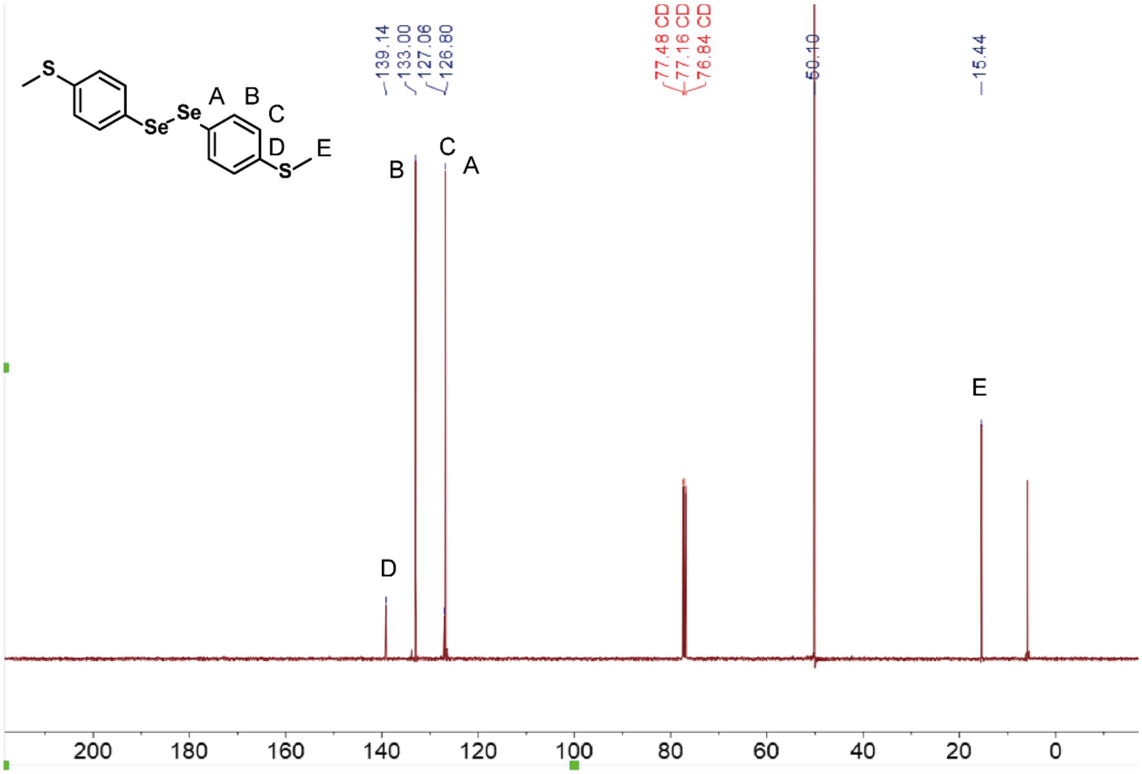

^{77}Se NMR (101 MHz, $CDCl_3$) of 1,2-bis(4-thiomethylphenyl) diselenide

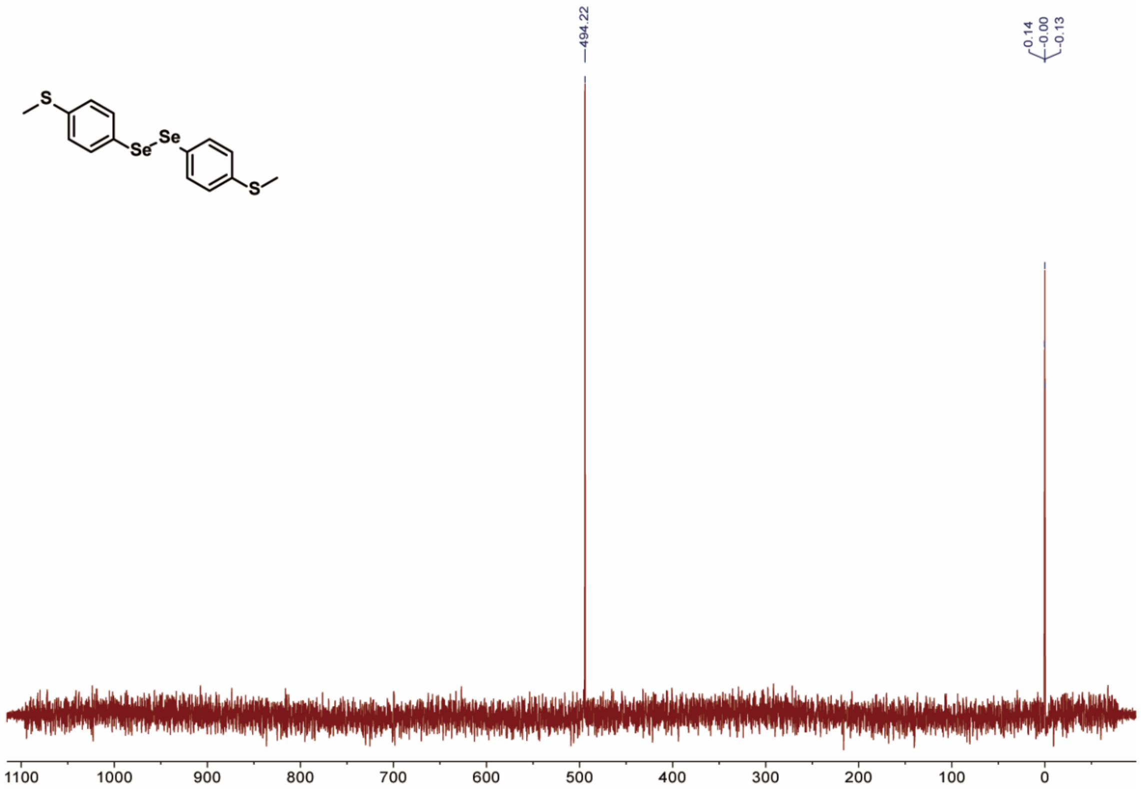

^1H NMR (400 MHz, $CDCl_3$) of 1,2-bis(2-naphthyl) diselenide

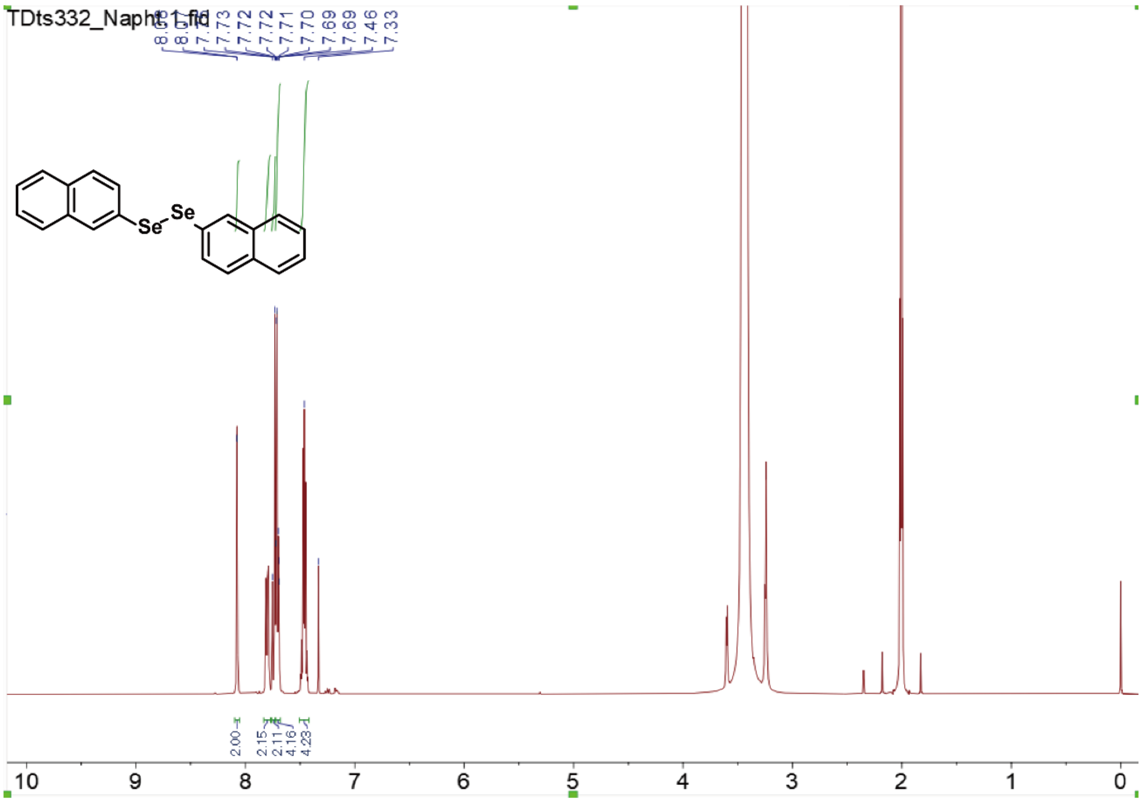

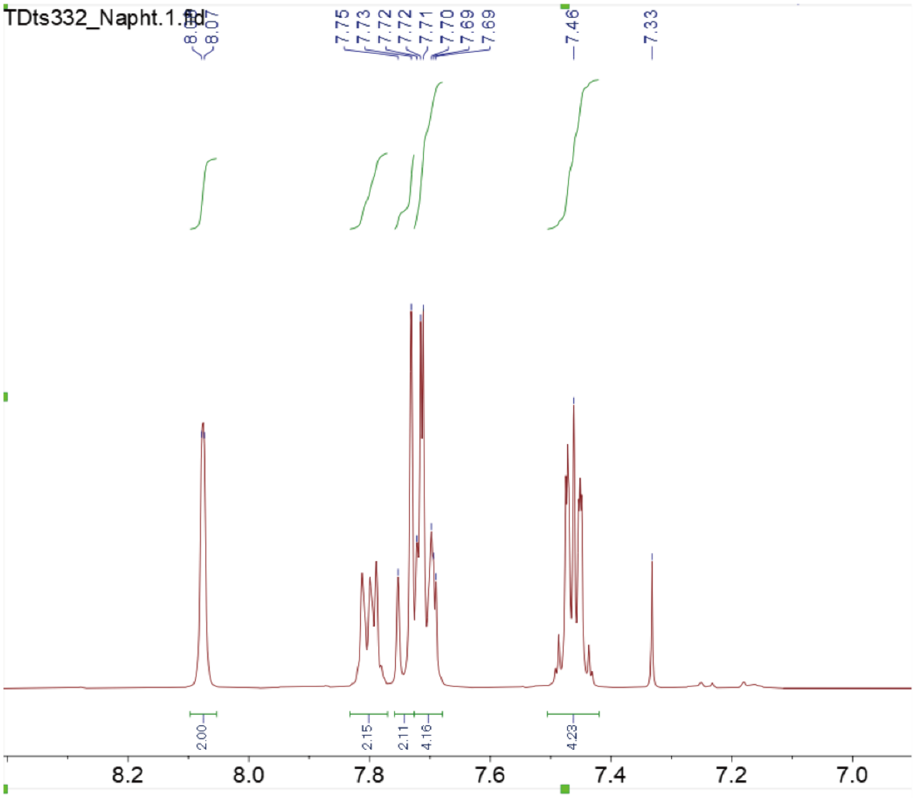

^{13}C NMR (101 MHz, $CDCl_3$) of 1,2-bis(2-naphthyl) diselenide

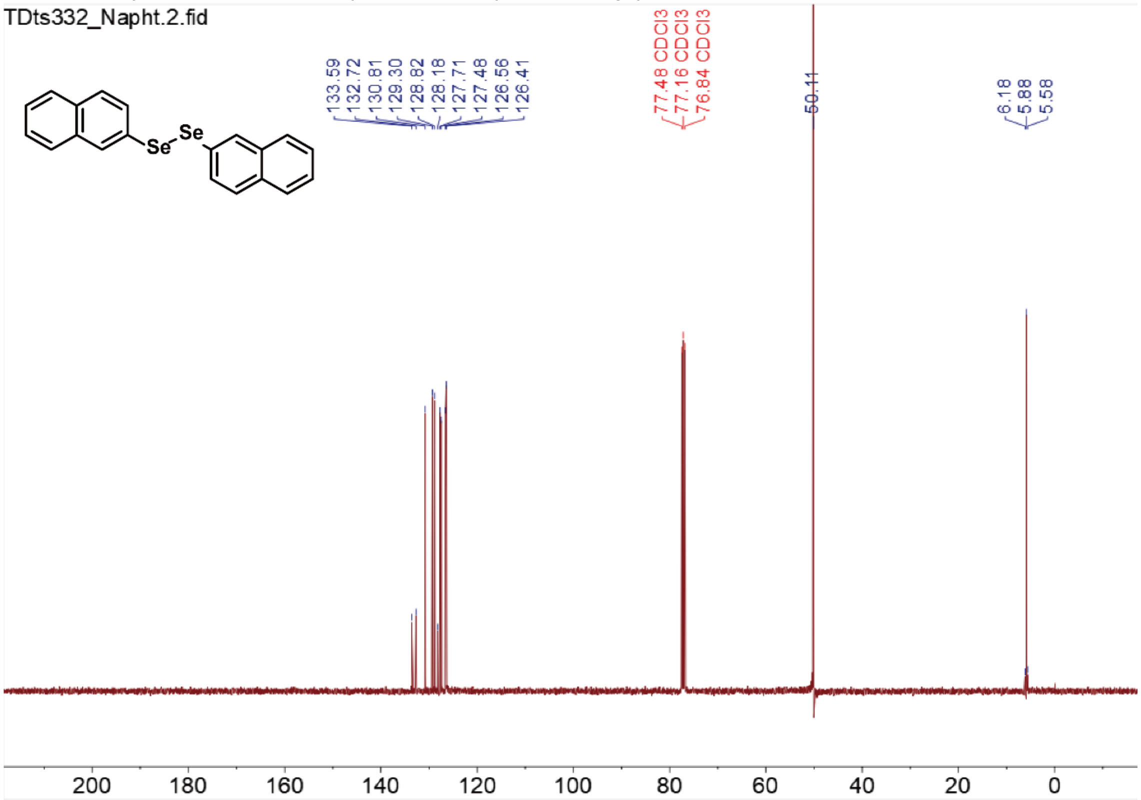

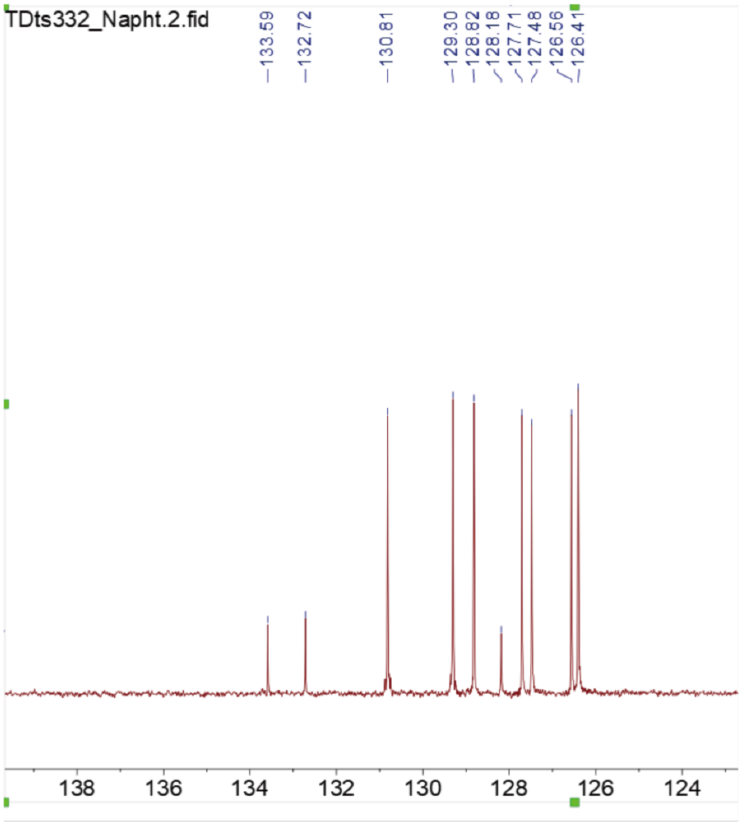

^{77}Se NMR (101 MHz, $CDCl_3$) of 1,2-bis(2-naphthyl) diselenide

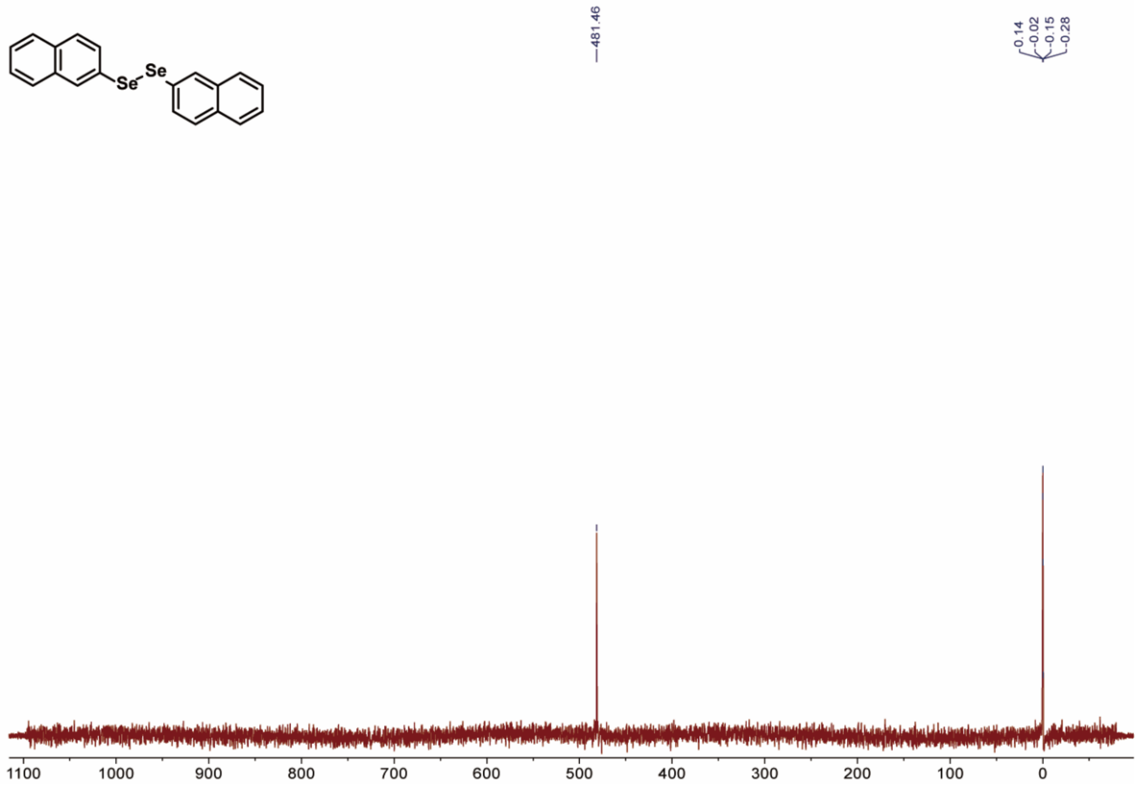

4. References

(1) Kubelka, P.; Munk, F. An Article on Optics of Paint Layers. *Z. Technol. Phys.* **1931**, *12*, 593.

(2) Sheldrick, G. M. SHELXT – Integrated Space-Group and Crystal-Structure Determination. *Acta Cryst A* **2015**, *71* (1), 3–8. https://doi.org/10.1107/S2053273314026370.

(3) Sheldrick, G. M. Crystal Structure Refinement with SHELXL. *Acta Cryst C* **2015**, *71* (1), 3–8. https://doi.org/10.1107/S2053229614024218.

(4) Müller, P. Practical Suggestions for Better Crystal Structures. *Crystallography Reviews* **2009**, *15* (1), 57–83. https://doi.org/10.1080/08893110802547240.

(5) de Mello, J. C.; Wittmann, H. F.; Friend, R. H. An Improved Experimental Determination of External Photoluminescence Quantum Efficiency. *Adv. Mater.* **1997**, *9* (3), 230–232. https://doi.org/10.1002/adma.19970090308.

(6) Kresse, G.; Furthmüller, J. Efficient Iterative Schemes for Ab Initio Total-Energy Calculations Using a Plane-Wave Basis Set. *Phys. Rev. B* **1996**, *54* (16), 11169–11186. https://doi.org/10.1103/PhysRevB.54.11169.

(7) Kresse, G.; Furthmüller, J. Efficiency of Ab-Initio Total Energy Calculations for Metals and Semiconductors Using a Plane-Wave Basis Set. *Computational Materials Science* **1996**, *6* (1), 15–50. https://doi.org/10.1016/0927-0256(96)00008-0.

(8) Kresse, G.; Hafner, J. Ab Initio Molecular Dynamics for Liquid Metals. *Phys. Rev. B* **1993**, *47* (1), 558–561. https://doi.org/10.1103/PhysRevB.47.558.

(9) Kresse, G.; Joubert, D. From Ultrasoft Pseudopotentials to the Projector Augmented-Wave Method. *Phys. Rev. B* **1999**, *59* (3), 1758–1775. https://doi.org/10.1103/PhysRevB.59.1758.

(10) Perdew, J. P.; Burke, K.; Ernzerhof, M. Generalized Gradient Approximation Made Simple. *Phys. Rev. Lett.* **1996**, *77* (18), 3865–3868. https://doi.org/10.1103/PhysRevLett.77.3865.

(11) Blöchl, P. E. Projector Augmented-Wave Method. *Phys. Rev. B* **1994**, *50* (24), 17953–17979. https://doi.org/10.1103/PhysRevB.50.17953.

(12) Grimme, S.; Antony, J.; Ehrlich, S.; Krieg, H. A Consistent and Accurate Ab Initio Parametrization of Density Functional Dispersion Correction (DFT-D) for the 94 Elements H-Pu. *The Journal of Chemical Physics* **2010**, *132* (15), 154104. https://doi.org/10.1063/1.3382344.

(13) Grimme, S.; Ehrlich, S.; Goerigk, L. Effect of the Damping Function in Dispersion Corrected Density Functional Theory. *Journal of Computational Chemistry* **2011**, *32* (7), 1456–1465. https://doi.org/10.1002/jcc.21759.

(14) Neese, F.; Wennmohs, F.; Becker, U.; Riplinger, C. The ORCA Quantum Chemistry Program Package. *The Journal of Chemical Physics* **2020**, *152* (22), 224108. https://doi.org/10.1063/5.0004608.

(15) Becke, A. D. A New Mixing of Hartree–Fock and Local Density-Functional Theories. *The Journal of Chemical Physics* **1993**, *98* (2), 1372–1377. https://doi.org/10.1063/1.464304.

(16) Lee, C.; Yang, W.; Parr, R. G. Development of the Colle-Salvetti Correlation-Energy Formula into a Functional of the Electron Density. *Phys. Rev. B* **1988**, *37* (2), 785–789. https://doi.org/10.1103/PhysRevB.37.785.

(17) Vosko, S. H.; Wilk, L.; Nusair, M. Accurate Spin-Dependent Electron Liquid Correlation Energies for Local Spin Density Calculations: A Critical Analysis. *Can. J. Phys.* **1980**, *58* (8), 1200–1211. https://doi.org/10.1139/p80-159.

(18) Weigend, F.; Ahlrichs, R. Balanced Basis Sets of Split Valence, Triple Zeta Valence and Quadruple Zeta Valence Quality for H to Rn: Design and Assessment of Accuracy. *Phys. Chem. Chem. Phys.* **2005**, *7* (18), 3297–3305. https://doi.org/10.1039/B508541A.

(19) Weigend, F. Accurate Coulomb-Fitting Basis Sets for H to Rn. *Phys. Chem. Chem. Phys.* **2006**, *8* (9), 1057–1065. https://doi.org/10.1039/B515623H.

(20) Hellweg, A.; Hättig, C.; Höfener, S.; Klopper, W. Optimized Accurate Auxiliary Basis Sets for RI-MP2 and RI-CC2 Calculations for the Atoms Rb to Rn. *Theor Chem Acc* **2007**, *117* (4), 587–597. https://doi.org/10.1007/s00214-007-0250-5.

(21) Reed, A. E.; Weinstock, R. B.; Weinhold, F. Natural Population Analysis. *The Journal of Chemical Physics* **1985**, *83* (2), 735–746. https://doi.org/10.1063/1.449486.
(22) Reich, H. J.; Renga, J. M.; Reich, I. L. Organoselenium Chemistry. Conversion of Ketones to Enones by Selenoxide Syn Elimination. *J. Am. Chem. Soc.* **1975**, *97* (19), 5434–5447. https://doi.org/10.1021/ja00852a019.
(23) Rode, K.; Palomba, M.; Ortgies, S.; Rieger, R.; Breder, A. Aerobic Allylation of Alcohols with Non-Activated Alkenes Enabled by Light-Driven Selenium-π-Acid Catalysis. *Synthesis* **2018**, *50* (19), 3875. https://doi.org/10.1055/s-0037-1609938.
(24) Li, Z.; Ke, F.; Deng, H.; Xu, H.; Xiang, H.; Zhou, X. Synthesis of Disulfides and Diselenides by Copper-Catalyzed Coupling Reactions in Water. *Org. Biomol. Chem.* **2013**, *11* (18), 2943. https://doi.org/10.1039/c3ob40464a.
(25) Hyugano, T.; Liu, S.; Ouchi, A. Facile Photochemical Transformation of Alkyl Aryl Selenides to the Corresponding Carbonyl Compounds by Molecular Oxygen: Use of Selenides as Masked Carbonyl Groups. *J. Org. Chem.* **2008**, *73* (22), 8861–8866. https://doi.org/10.1021/jo801730j.
(26) Paritmongkol, W.; Sakurada, T.; Lee, W. S.; Wan, R.; Müller, P.; Tisdale, W. A. Size and Quality Enhancement of 2D Semiconducting Metal–Organic Chalcogenolates by Amine Addition. *J. Am. Chem. Soc.* **2021**, *143* (48), 20256–20263. https://doi.org/10.1021/jacs.1c09106.
